# Supplementary material for: Development of oil-based gels as versatile drug delivery systems for pediatric applications
Source: Sci Adv. 2022 May 27;8(21):eabm8478. doi: 10.1126/sciadv.abm8478 (PMC9140966; doi:10.1126/sciadv.abm8478)
Supplement: 20220527-1 [file sciadv.abm8478.v1.pdf]

LIFE SCIENCES

Development of oil-based gels as versatile drug delivery systems for pediatric applications

Copyright © 2022 The Authors, some rights reserved; exclusive licensee American Association for the Advancement of Science. No claim to original U.S. Government Works. Distributed under a Creative Commons Attribution License 4.0 (CC BY).

Ameya R. Kirtane[1,2]†, Christina Karavasili[1]†, Aniket Wahane[1]†, Dylan Freitas[2], Katelyn Booz[3], Dao Thi Hong Le[1,4], Tiffany Hua[1], Stephen Scala[1,5]‡, Aaron Lopes[1,2], Kaitlyn Hess[1], Joy Collins[1,2], Siddartha Tamang[1], Keiko Ishida[1], Johannes L. P. Kuosmanen[1], Netra Unni Rajesh[1,6], Nhi V. Phan[1], Junwei Li[1,2], Annlyse Krogmann[3], Jochen K. Lennerz[7], Alison Hayward[1,2,8], Robert Langer[1,9], Giovanni Traverso[1,2,9]*

Administering medicines to 0- to 5-year-old children in a resource-limited environment requires dosage forms that circumvent swallowing solids, avoid on-field reconstitution, and are thermostable, cheap, versatile, and taste masking. We present a strategy that stands to solve this multifaceted problem. As many drugs lack adequate water solubility, our formulations used oils, whose textures could be modified with gelling agents to form "oleogels." In a clinical study, we showed that the oleogels can be formulated to be as fluid as thickened beverages and as stiff as yogurt puddings. In swine, oleogels could deliver four drugs ranging three orders of magnitude in their water solubilities and two orders of magnitude in their partition coefficients. Oleogels could be stabilized at 40°C for prolonged durations and used without redispersion. Last, we developed a macrofluidic system enabling fixed and metered dosing. We anticipate that this platform could be adopted for pediatric dosing, palliative care, and gastrointestinal disease applications.

INTRODUCTION

Children under the age of 5 years are vulnerable to a host of treatable and preventable childhood diseases (*1*). This is especially observed for children in countries with a low sociodemographic index, who experience a much higher disease rate and resultant mortality (*2*, *3*). Specifically, Angola, Central Africa Republic, Chad, Mali, Nigeria, Sierra Leone, and Somalia account for 20% of the world's under-five mortality (*2*). Hence, there is a critical need to identify practical life-saving interventions and then make them available in countries that remain susceptible to high childhood mortality.

Drugs can be very effective in reducing the impact of childhood diseases. A notable example of this was a recent study that showed that communities in which children were treated with a twice-yearly broad-spectrum antibiotic, azithromycin, had a significantly lower mortality rate in comparison to communities in which children received placebo (*4*). Because of the ease and low cost of manufacturing, drugs are typically formulated as tablets. However, children can have difficulties swallowing tablets. In a survey carried out in the United States, more than 50% of parents indicated that their child had difficulty swallowing standard-sized pills (*5*). Administration of tablets to children under the age of 36 months can cause choking; if the staff or parent administering the tablet does not know how to help, then the consequence can be fatal (*6*). In two 2006 field studies of vaccine and deworming campaigns in Rwanda and Madagascar, the choking incidence was 1 to 3% (*7*). Although these occurrences are rare, they can have a substantial and important impact in mass drug administration campaigns.

To address the challenges associated with swallowing of tablets, several innovative strategies have been devised—both at the level of formulation development in the pharmaceutical industry and as stopgap practices applied in the field. The formulation of drugs in aqueous solutions and syrups is one of the oldest and most effective means of drug administration to children. Suspensions are also liquid dosage forms, and these are suitable for water-insoluble drugs that cannot be dissolved in solutions/syrups. The particle size in suspensions must be carefully controlled to prevent aggregation, ensure easy dispersion, and enable efficient drug absorption. Long-term instability and poor drug absorption can be alleviated by using spray-dried nanoparticles (*8*–*10*) that are dispersed right before administration. As these are in liquid form for a short period, settling does not occur, although a clean source of water may present on the ground constraints. In addition, because of their small particle size, the rate of dissolution is improved, ultimately aiding in drug absorption. Despite the availability of these pediatric-friendly dosage forms, not all drugs are formulated as such. In the absence of an appropriate dosage form for children, a common practice in the field involves health care professionals crushing the tablet, dispersing it in water, and administering the suspension to the child. Hence, there are a range of strategies for administering medicines to children.

While these strategies have several advantages, they present some key limitations. Solutions and syrups can be applied to only water-soluble drugs and hence exclude most drugs, which are typically water insoluble. Ethanol is a commonly used cosolvent in oral formulations, but its use in pediatric formulations is restricted. The U.S. Food and Drug Administration (FDA) allows no more

[1]David H. Koch Institute for Integrative Cancer Research and Department of Chemical Engineering, Massachusetts Institute of Technology, Cambridge, MA 02139, USA. [2]Division of Gastroenterology, Brigham and Women's Hospital, Harvard Medical School, Boston, MA 02115, USA. [3]Sensory Spectrum Inc., New Providence, NJ 07974, USA. [4]Swiss Federal Institute of Technology (ETH), Zurich 8092, Switzerland. [5]Stonehill College, North Easton, MA 02357, USA. [6]University of Toronto, Toronto, ON, Canada. [7]Department of Pathology, Center for Integrated Diagnostics, Massachusetts General Hospital, Harvard Medical School, Boston, MA 02114, USA. [8]Division of Comparative Medicine, Massachusetts Institute of Technology, Cambridge, MA 02139, USA. [9]Department of Mechanical Engineering, Massachusetts Institute of Technology, Cambridge, MA 02139, USA.
*Corresponding author. Email: cgt20@mit.edu, ctraverso@bwh.harvard.edu
†These authors contributed equally to this work.
‡Present address: Boeing Company, Herndon, VA 20170, USA.

than 0.5% ethanol in over-the-counter drug products for children under 6 years of age (*11*, *12*). Suspensions (preformed or those produced at the time of dosing) will require the patient, guardian, or health care provider to mix (shake) the formulation before use. Failure to do so can result in inaccurate dosing (*13*). Hence, the success of these dosage forms heavily rests on the habits/training of the user. Last, crushing tablets, although a routine, may not be advisable (*14*) as the drug may not be evenly distributed in the tablet (*15*) unless such validation is performed, which introduces variability in dosing.

In this project, our goal was to develop a dosage form that could be used to administer medicines to children especially in resource-limited settings. We adopt a formulation technique described in the field of molecular gastronomy that transforms oils into gels, known as oleogels (*16*). Oleogels are oil-structured systems that are made from a gelling agent added to an edible oil. Oleogels are formed by heating, shearing, and cooling the hydrophobic system, resulting in the generation of a three-dimensional (3D) network of crystalline particles, self-assembled fibers, or polymers entrapping the liquid oil (*17*). This technique has been used to increase the melting temperature of oils to render foods such as chocolates heat resistant (*18*). Conversion of vegetable oils to gels also allows for substitution of animal fats (*19*) to satisfy dietary restrictions. Moreover, using gels instead of liquid oils prevents oil separation from foods, such as cupcakes, aiding long-term storage and improving consumer satisfaction (*20*). While the use of oils for delivering drugs has been widely explored, there are only few studies that use oleogels for the oral delivery of lipophilic compounds (*21*–*24*). Moreover, the utility of oil-based vehicles in delivery of water-soluble compounds has not been widely studied. We show here that the oleogel formulation can be tailored for administration of medicines to children in low sociodemographic index countries, which we realize presents several unique challenges. In that, the oleogels can be made to remain stable at high temperatures for prolonged durations, can be used to administer drugs with a range of physicochemical properties, and can be dispensed using metered dosing systems without the need for mixing/reconstitution. We believe that our approach overcomes several challenges associated with formulation design and patient satisfaction and can be used in the care of a highly vulnerable, yet often overlooked, patient population.

RESULTS

Analysis of the World Health Organization model list of essential medicines for children

We were interested in defining the general availability of different dosage form types for the most used drugs in pediatric care. Hence, we used the World Health Organization (WHO) model list of essential medicines for children (*25*), a minimum list of medicines needed by a basic health care system. We categorized these essential medications on the basis of their target disease. Drug products intended to treat infectious diseases (44%), neurological diseases (10%), and pain management (8.4%) formed the bulk of the list (Fig. 1A). Other notable categories included medications for cancer and cardiovascular diseases, each of which contained more than 5% of the medications on the list.

We then analyzed the various drug categories among the top three disease areas (Fig. 1B). Among infectious diseases, antibacterials were the most listed drug products and constituted about half of the anti-infectives. Other common anti-infectives included antiviral and antimalarial drugs. Anticonvulsant drug products formed most of those listed for the management of neurological diseases. Drug products that could be used for pain management had comparable numbers to each of the following: opioid analgesics, nonsteroidal and nonopioid analgesics, and local anesthetics.

Patient acceptability of drug products depends, in part, on the route of administration. Hence, we studied the most popular routes of administration for the drug products in this list. Expectedly, about 60% of the drug products were intended to be used orally (Fig. 1C). Other common routes of administration included parenteral (27.2%), topical (5.8%), and rectal (2.3%).

About 72% of the oral products were intended to be consumed as solids (Fig. 1D). This did not include granules, powder for reconstitution, crushable tablets, or dispersible tablets, which we categorized as liquids because of the change in their physical form right before administration. Roughly 90% of the solid dosage forms were tablets (immediate and sustained release) and capsules—dosage forms that can be challenging for children to swallow. These data confirmed to us that there is a critical need to develop dosage forms that are child friendly.

Concept and design of oleogels

With the goal of pediatric dosage forms that can be applied in resource-limited settings, we aimed to design systems with the following characteristics: (i) compatible with hydrophobic and hydrophilic drugs, (ii) does not require the patient to swallow a large solid, (iii) does not require reconstitution, (iv) safe to use in children, (v) stable under extreme temperatures/humidity, (vi) exhibits favorable pharmacokinetics, and (vii) compatible with oral and rectal dosing. To achieve this, we developed a drug delivery system using oleogels. Oleogels are made predominantly of cooking oils whose consistency is adjusted by adding gelling agents. To dissolve drugs at high concentrations, we supplemented oleogels with surfactants, used in both the food and pharmaceutical industry. In this study, our goal was to define a range of effects each component of the oleogels could have on the final formulation. We then developed proof-of-concept formulations for four anti-infectives—azithromycin, lumefantrine, praziquantel, and moxifloxacin—and characterized their pharmacokinetics upon oral and rectal administration in pigs.

Identification of gelling agents for the formation of oleogels

Converting liquids into gels can alter the consistency and mouthfeel of a product, which can affect consumer acceptability (*26*). Numerous approaches have been described to form aqueous gels. These include use of linear water-soluble polymers (*27*, *28*), cross-linking of water-soluble polymers (*29*–*31*), temperature-based (*32*) and pH-based (*33*) assembly of polymers, and use of self-assembling nanoparticles (*34*). However, much less is known about the formation of oil-based gels. Here, we set out to identify ingredients that enable the formation of gels in oils. Candidate gelling agents were added to the oil and heated past their melting point to yield a clear liquid. The molten mixture was cooled to room temperature to form the gel. The formulation was considered a gel if it was unable to flow upon inversion. The minimum concentration at which a gelling agent formed a gel was considered its critical gelling concentration.

We first evaluated the ability of linear fatty acids to form oleogels (Fig. 2A). Lauric acid (C12), the smallest linear fatty acid tested, formed gels at a concentration of 10% (w/w). Long-chain fatty

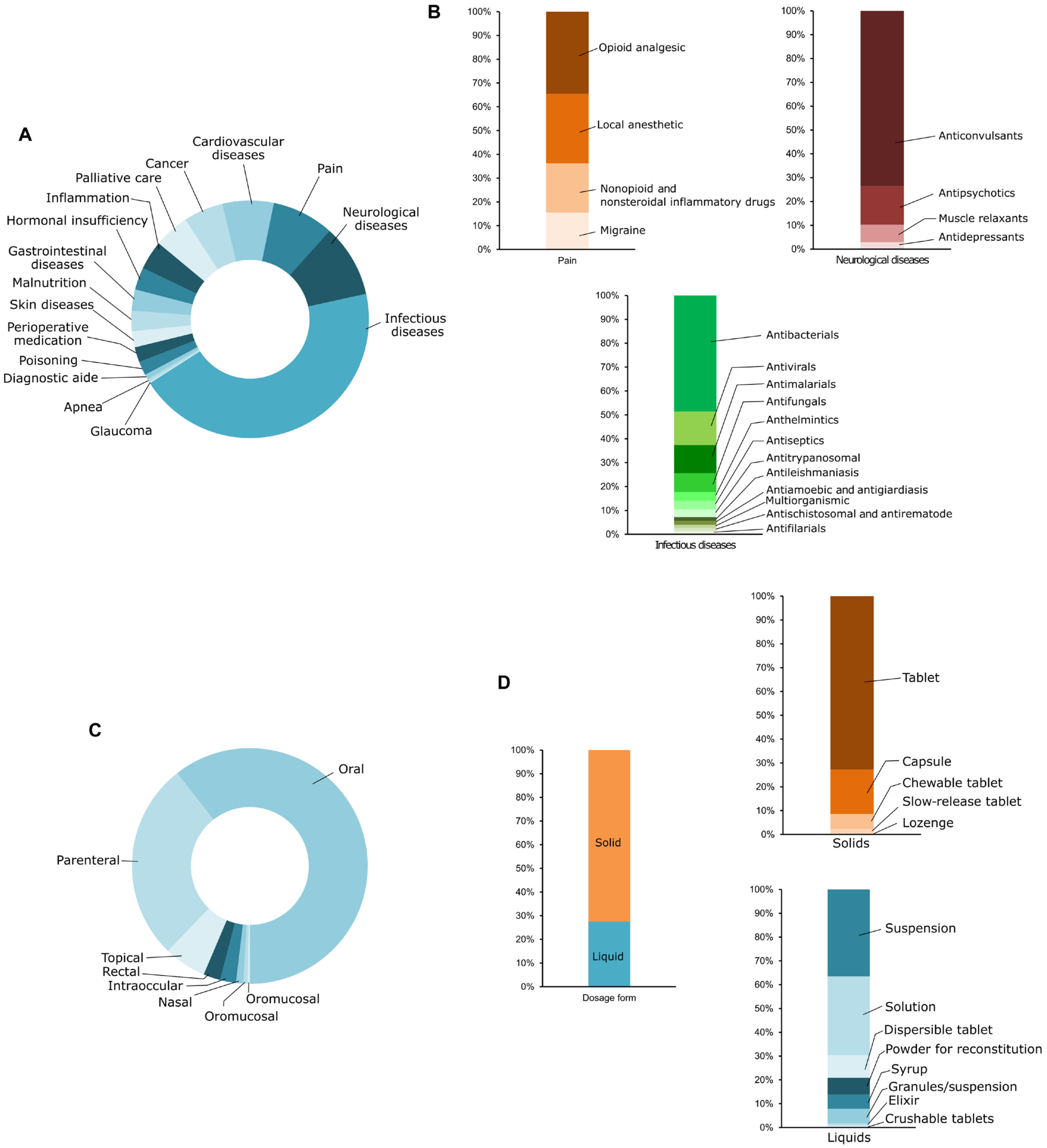

Fig. 1. Analysis of the WHO's essential medicine list for children. (**A**) Relative proportion of drugs classified by disease area. (**B**) Detailed analysis of the relative proportion of drugs used for the treatment of pain and neurological and infectious diseases. (**C**) Routes of administration of drugs used for the treatment of infectious diseases. (**D**) Commonly used dosage forms for oral administration of anti-infective drugs.

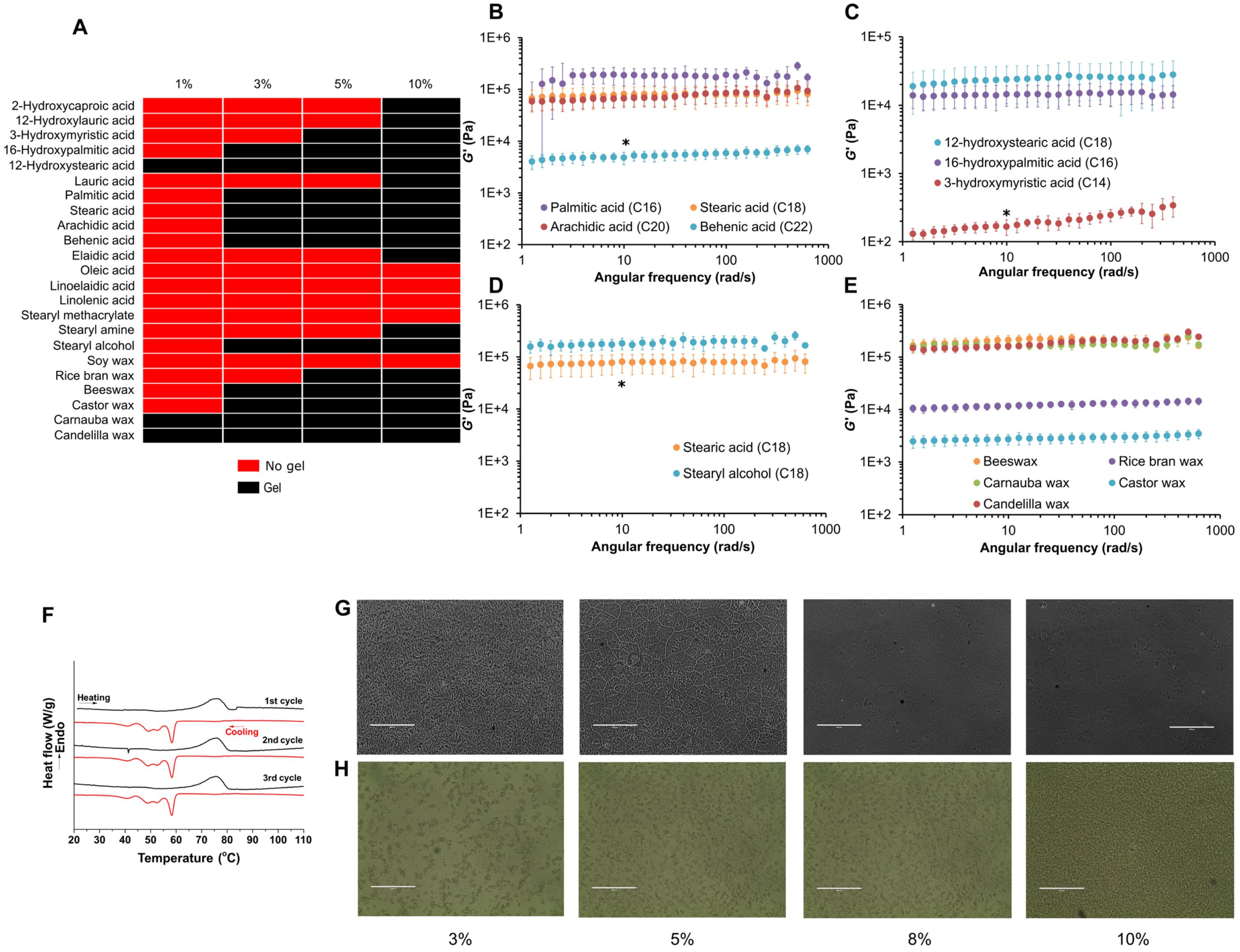

Fig. 2. Physical characterization of oleogels. (**A**) Identification of gelling agents using an inversion assay. (**B**) Comparison of rheological properties of oleogels prepared using saturated fatty acids with differing carbon chain lengths. *$P < 0.05$; one-way analysis of variance (ANOVA), post hoc Bonferroni versus palmitic acid at angular frequency = 10 rad/s. Data are represented as means ± SD; $n = 3$. (**C**) Comparison of rheological properties of oleogels prepared using saturated hydroxyl fatty acids. Data are represented as means ± SD; $n = 3$. *$P < 0.05$; Student's t test versus 16-hydroxypalmitic acid at angular frequency = 10 rad/s. (**D**) Rheological analysis of oleogels prepared using stearic acid and stearyl alcohol. Data are represented as means ± SD; $n = 3$. *$P < 0.05$; Student's t test at angular frequency = 10 rad/s. (**E**) Rheological analysis of oleogels prepared using waxes. Data are represented as means ± SD; $n = 3$. (**F**) DSC analysis of oleogel containing rice bran wax as the gelling agent. Optical microscopy analysis of oleogels prepared using (**G**) hydroxystearic acid and (**H**) rice bran wax. Scale bars, 400 μm (G) and 200 μm (H).

acids, such as palmitic (C16), stearic (C18), arachidic (C20), and behenic acid (C22), could form gels at lower concentrations [3% (w/w)]. When comparing hydroxystearic acid and stearic acid, we observed that the hydroxy fatty acid could form gels at a lower concentration than the fatty acid.

Next, we assessed gel formation with unsaturated fatty acids. We compared the gelling capacity of two isomers of monounsaturated octadecanoic acid, namely, elaidic acid and oleic acid. The cis-isomer (oleic acid) was not capable of forming gels in the concentration range that we tested, while the trans-isomer (elaidic acid) formed gels at a concentration of 10% (w/w). At the concentration range used in our study, polyunsaturated fatty acids were unable to form gels.

To understand the effect of the terminal group, we compared the gel formation capacity of stearic acid, stearyl amine, stearyl alcohol, and stearyl methacrylate. The critical gelling concentration of stearyl alcohol was the same as that of stearic acid [3% (w/w)]. The amine formed gels at high concentrations [10% (w/w)], while the methacrylate was unsuccessful at forming gels at the concentrations tested.

Last, we studied gel formation using six waxes that are widely used in the food industry as bland texture-manipulating agents or as coating agents (*35*–*37*). Carnauba wax and candelilla wax were the most effective gelling agents [1% (w/w)]. Soy wax was unable to form gels at the highest concentration used in these studies. Rice bran wax and beeswax had intermediate critical gelling concentrations. In summary, the ability to form oil-based gels was dependent on molecular weight, degree of unsaturation, chirality, and the functional groups of the various gelling agents.

Physical characterization of oleogels

We were interested in understanding the effect of gelling agent on the rheological properties of the oleogel. We first tested oleogels synthesized using saturated fatty acids of various chain lengths (Fig. 2B). In this group, oleogels formed with palmitic acid (C16) had the highest gel strength. Oleogels prepared with fatty acids with longer chain lengths, such as arachidic acid (C20), had nearly one-quarter of the gel strength of palmitic acid containing oleogels [46 ± 22 kPa, n = 3 versus 187 ± 71 kPa, n = 3, at angular frequency = 10 rad/s; *P < 0.05, one-way analysis of variance (ANOVA)]. The G' value for the oleogels prepared with behenic acid, the largest fatty acid in our study (C22), was nearly 40-fold lower than the oleogels prepared with palmitic acid (C16) (4.8 ± 1.3 kPa, n = 3 versus 187 ± 71 kPa, n = 3, at angular frequency = 10 rad/s; *P < 0.05, one-way ANOVA with post-hoc Bonferroni).

We then investigated the gel strength of oleogels prepared using hydroxy fatty acids (Fig. 2C) and found a near opposite trend. In that, the smallest hydroxy fatty acid tested in our studies [3-hydroxymyristic acid (C14)] formed the weakest gels, while larger hydroxy fatty acids formed stronger gels (0.17 ± 0.42 kPa, n = 3 for 3-hydroxymyristic acid versus 14.4 ± 4.7 kPa, n = 3 for 16-hydroxypalmitic acid, at angular frequency = 10 rad/s; *P < 0.05, Student's t test). Note that the location of the hydroxyl group is different across the hydroxy fatty acids.

Next, we compared the effect of terminal functional groups on the gel strength of the oleogels. The gel strength of the stearyl alcohol–based oleogels was nearly twice that of stearic acid–based oleogels (182.2 ± 45.3 kPa, n = 3 versus 81.0 ± 32.1 kPa, n = 3, at angular frequency = 10 rad/s; *P < 0.05, Student's t test) (Fig. 2D).

Last, we compared the rheological characteristics of oleogels prepared with five different waxes (Fig. 2E). We observed that the wax-based oleogels had G' values that spanned two orders of magnitude. Beeswax, carnauba wax, and candelilla wax formed the strongest gels with comparable G' values. The G' value of the gels prepared with castor wax was the lowest, while gels prepared with rice bran wax had intermediate G' value.

We used differential scanning calorimetry (DSC) to evaluate the thermal behavior of the oleogels prepared with rice bran wax. The thermogram of the rice bran wax–based oleogels showed a characteristic melting endothermic peak with an onset at 75°C and a sequential crystallization upon cooling with an onset at 58°C (Fig. 2F). This highlights the relative heat-resistant behavior of the system, which can be an asset in the absence of cold supply chains. In addition, the melting-crystallization transition of the rice bran wax–based oleogels is a reversible process, indicating that the system can recover to its initial state after exposure to increased temperatures.

Using light microscopy, we studied the microstructures of the oleogels prepared using rice bran wax and 12-hydroxystearic acid. Oleogels containing low concentrations of 12-hydroxystearic acid exhibited branched dendritic crystals, while those made with higher concentrations of 12-hydroxystearic acid exhibited a rosette-like crystal morphology (Fig. 2G). Rice bran wax–based oleogels had a fibrous morphology, forming a dendritic interconnected network with crystal size ranging between 10 and 20 μm (Fig. 2H). Crystal size was positively correlated with rice bran wax concentration. This was attributed to the higher available amount of crystalline material facilitating crystal growth, as previously reported (*38*).

Our studies showed that gelling agents formed expansive dendritic microstructures to yield oleogels. The strength of these gels could be tailored to fit consumer preference or application needs by using different gelling agents.

Measuring drug solubility in oil-solubilizer mixtures

Dissolution is the rate-limiting step in the absorption of drugs belonging to class II of the Biopharmaceutical Classification System (BCS). Moreover, it is one of the rate-limiting steps in the absorption of BCS class IV drugs. Hence, we were interested in designing a drug delivery system that contained the drug in solution. To design such a formulation, we first sought to understand how choice of oil affected drug solubility. We chose nine plant-based oils for these studies (Fig. 3A). The major components of all oils were mono- and di-unsaturated 18-carbon fatty acids (*39*). In addition, the oils contained varying levels of other fatty acids and sterols, which provided us with a diverse formulation library. To further increase diversity, we decided to mix the oils with 11 solubilizing agents (Fig. 3B). Solubilizing agents were predominantly fatty acid esters of di- and tri-alcohols. All solubilizing agents have been previously used in foods and drug products approved by the FDA and were used at a concentration comparable to those used in FDA-approved products. Because anti-infectives are the largest drug class on the WHO model drug list, we decided to conduct our solubility studies with three anti-infectives—azithromycin, praziquantel, and lumefantrine (Fig. 3, C to E).

The solubility of azithromycin in the oils was ~6 to 10 mg/g (Fig. 3C). The addition of the solubilizers Lauroglycol 90 and Labrafac lipophile led to a slight decrease in solubility. All other solubilizers increased solubility to varying degrees. The maximal increase in solubility occurred with Capryol 90 [31.7 ± 1.6 mg/g (olive oil and Capryol 90) versus 8.9 ± 0.55 mg/g (olive oil alone), n = 3; P < 0.05, one-way ANOVA with post-hoc Bonferroni] and Peceol [22.5 ± 0.8 mg/g (olive oil + Peceol) versus 8.9 ± 0.55 mg/g (olive oil alone), n = 3; P < 0.05, one-way ANOVA with post-hoc Bonferroni]. In contrast, the solubility of azithromycin in water is ~0.2 mg/g (*40*), nearly 1/100th that of our top formulations.

The solubility of praziquantel in the various oil-solubilizer formulations is shown in Fig. 3D. Praziquantel was soluble up to 5 to 7 mg/g in most oils. Its solubility in sunflower oil was twice as high as its solubility in the rest of the oils (17.8 ± 2.2 mg/g for sunflower oil versus 7.2 ± 3.9 mg/g for all oils, n = 3; P < 0.05, one-sample t test). The addition of solubilizers led to an increase in solubility of praziquantel. Capryol 90 had maximal impact on drug solubility. Drug solubility in the mixture of Capryol 90 and flaxseed oil was 47.2 ± 3.6 mg/g, n = 3 (versus 6.1 ± 0.8 mg/g in flaxseed oil alone, n = 3; P < 0.05, two-sample t test). The solubility of praziquantel in the mixture of Capryol 90 and soybean oil was nearly half of that in the Capryol 90 + flaxseed oil mixture (23.6 ± 1.3 mg/g versus 47.2 ± 3.6 mg/g, n = 3; P < 0.05, two-sample t test). Hence, choice of both solubilizer and oil affected drug solubility.

The solubility of lumefantrine was generally comparable across all oils (>10 mg/g) (Fig. 3E). For 10 of 11 solubilizers, maximum solubility was observed when mixed with flaxseed oil. For example, the solubility of lumefantrine in the mixture of Labrafac lipophile and flaxseed oil was 26.6 ± 1.8 mg/g, n = 3. This was nearly double its average solubility in other formulations containing Labrafac lipophile (16.6 ± 3.8 mg/g; P < 0.05, one-sample t test). Notably, the solubility of lumefantrine observed in our top formulations was nearly 1000 times its reported water solubility [<20 μg/g (*41*)].

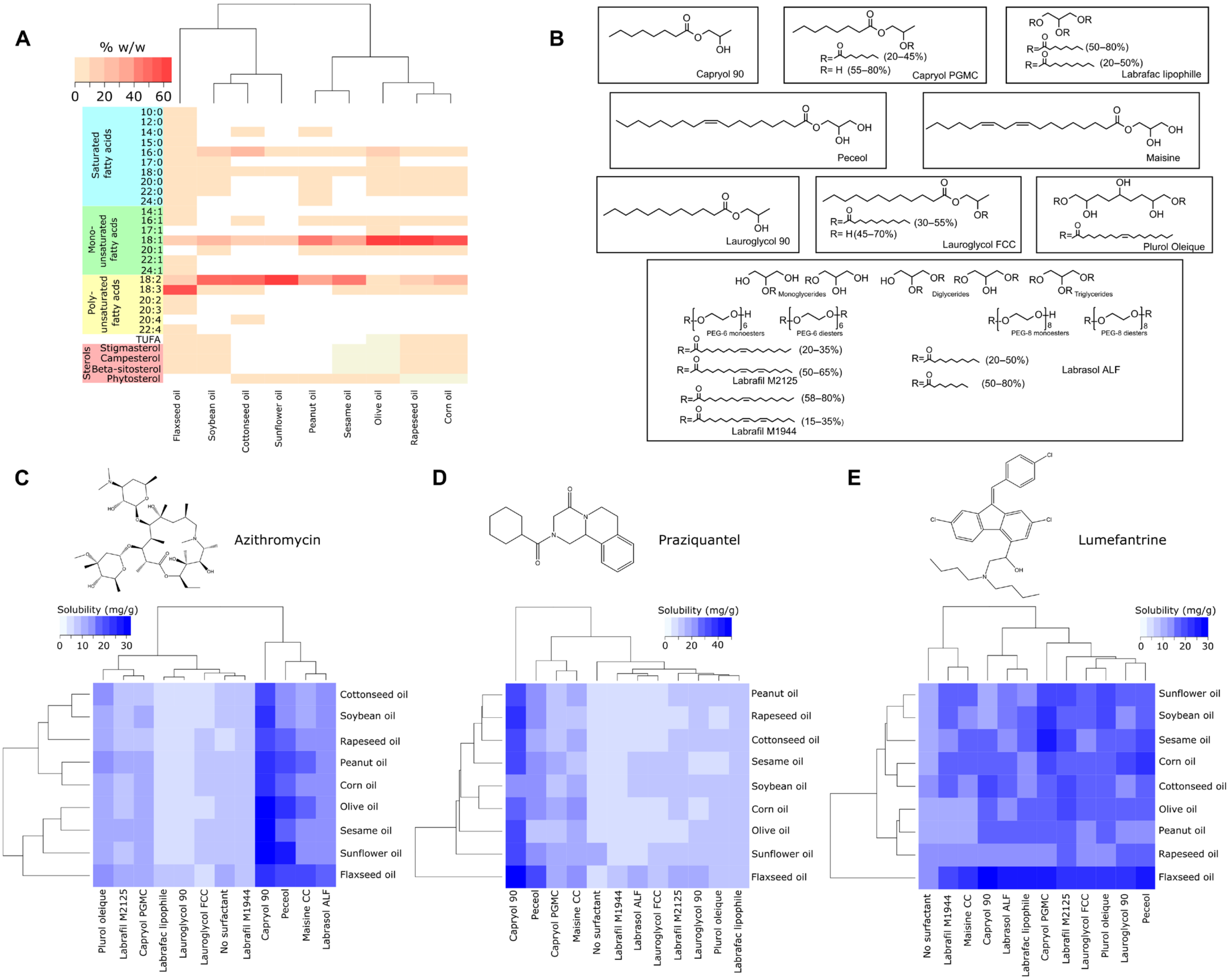

Fig. 3. Analysis of drug solubility in oil-solubilizer library. (**A**) Composition of oils used in this study. (**B**) Chemical structures of solubilizers used in this study. Solubility of (**C**) azithromycin, (**D**) praziquantel, and (**E**) lumefantrine in oil-solubilizer libraries as measured using high-performance liquid chromatography (HPLC). Mean of three samples is reported in the heatmap.

In vitro digestion of oleogels

We studied the digestion of oleogels in vitro in simulated salivary, gastric, and intestinal conditions (Fig. 4, A and B). Incubating the oleogel in simulated salivary and gastric conditions had minimal impact on the overall integrity of the gels. Following 2 hours of incubation in simulated gastric fluid (SGF), the gel remained phase-separated. In contrast, when the oleogel was placed in intestinal fluid, it rapidly disintegrated and emulsified (Fig. 4B), which was likely due to the presence of bile salts and surfactants.

Using cryo–transmission electron microscopy (cryo-TEM), we observed the lipolytic products generated during digestion (Fig. 4B, right). Different colloidal structures emerged with time. Intact oil droplets and rectangular particles were distinguished during the first 30 min of digestion. The presence of rectangular particles was identified only during the initial stage of lipolysis and may be the result of self-association of emulsifying components present in excess in the medium (*42*). At 60 min, the size of the oil droplets was between 30 and 150 nm. Digestion proceeded as mild “exfoliation” of the oil droplets, resulting in the formation of lamellar structures with high periodicity. Last, after 120 min, bilamellar and unilamellar vesicles coexisting with oil droplets and micelles were observed.

We then measured the amount of drug released in the various media (Fig. 4C). Consistent with our visual observations, drug release was maximal in the simulated intestinal fluid. The fraction of praziquantel present in the aqueous phase during the simulated gastric digestion reached its maximum level, 10% of the total drug content, within 30 min and remained at this plateau until the end of the 2-hour digestion process. The amount of praziquantel transferred to the aqueous phase of the simulated intestinal fluid was considered as a bioaccessible fraction of the drug. The bioaccessible fraction reached 51% at 15 min. A moderate decrease in praziquantel concentration in the aqueous phase was observed after 15 min from

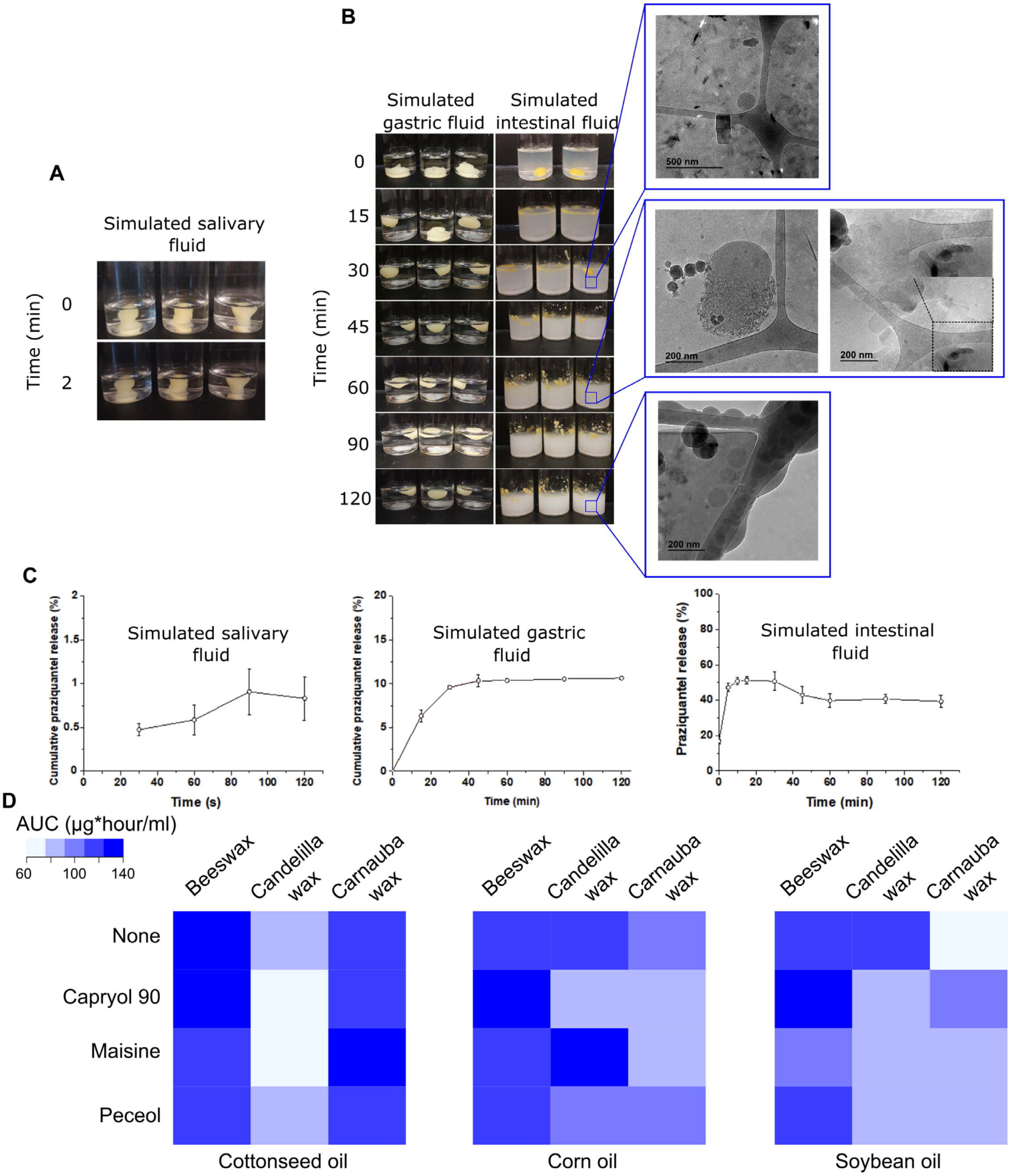

Fig. 4. In vitro digestion and bioaccessibility studies. (**A**) Image of oleogel suspended in simulated salivary fluid. (**B**) Images of oleogels in simulated gastric fluid and simulated intestinal fluid (fasted state). Insets on the right indicate cryo–transmission electron microscopy (cryo-TEM) images of the intermediate phase in simulated intestinal fluid at those times. (**C**) HPLC quantification of release of praziquantel from oleogels in simulated salivary, gastric, and intestinal fluids. Data are represented as means ± SD; $n = 3$ (**D**) Release of azithromycin from 36 oleogels was measured, and the area under the release curve was calculated. Heatmaps show mean AUC for three samples/formulation.

the initiation of lipolysis, possibly because of a decrease in the solubilization capacity of the digestion medium. The amount of praziquantel detected in the simulated salivary fluid (SSF) did not exceed 1% of the total drug content during the experiment.

In summary, in vitro drug release experiments indicated that the oleogel formulations would be mainly digested in the intestine, with minimum drug release in the salivary fluid. Formulations with such drug release profile may be favorable in reducing the bitter taste of many drug compounds; however, these effects may be drug specific, and clinical studies may be needed to test this further.

Effect of formulation components on drug release from oleogels

Oleogels contain three inactive ingredients—oil, solubilizer, and gelling agent. We were interested in understanding whether the choice of ingredients influenced drug release. We prepared azithromycin-loaded

oleogels using three oils (cottonseed oil, corn oil, and soybean oil) and three gelling agents (beeswax, candelilla wax, and carnauba wax) at four solubilizer conditions (no solubilizer, Capryol 90, Maisine, and Peceol). Drug release from oleogels was studied in simulated intestinal fluid and is plotted in figs. S1 to S3. We measured the area under the curve (AUC) for each drug release profile (Fig. 4D).

The choice of gelling agent had a critical impact on drug release, and this effect depended on the oil used in the formulation. Cottonseed oil–based oleogels made with carnauba wax had a higher AUC than those made with candelilla wax (126 ± 5 μg*hour/ml, n = 3 versus 58 ± 3 μg*hour/ml, n = 3, with Maisine; *P < 0.05, one-way ANOVA with post-hoc Bonferroni). However, for corn oil–based oleogels, use of candelilla wax led to higher AUC than carnauba wax (127 ± 1 μg*hour/ml, n = 3 versus 80 ± 5 μg*hour/ml, n = 3, with Maisine; *P < 0.05, one-way ANOVA with post-hoc Bonferroni). In both cottonseed oil– and corn oil–based oleogels, use of beeswax led to AUCs comparable to the better-performing gelling agent. In other words, beeswax containing oleogels performed well regardless of the oil. Beeswax has a lower melting point compared to the other waxes, a measure of lower intermolecular interaction. It is possible that lower intermolecular interaction in the gelling agent results in more ready disintegration of the gel and, consequently, higher release of drug in the aqueous media.

Perhaps unexpectedly, the use of solubilizers did not always produce higher AUCs. This phenomenon is most evident in the formulations made with soybean oil and candelilla wax. Here, introduction of solubilizers led to a ~35% reduction in the AUC as compared to the formulation devoid of the solubilizer (121 ± 1 μg*hour/ml, n = 3, for no solubilizer versus 78 ± 4, n = 3, for Capryol 90; *P < 0.05, one-way ANOVA with post-hoc Bonferroni). In select cases, introduction of a solubilizer did improve the AUC in comparison to the formulation lacking a solubilizer (98 ± 5 μg*hour/ml for soybean oil–carnauba wax–Capryol 90 formulation, n = 3 versus 74 ± 11 μg*hour/ml for soybean oil–carnauba wax–no solubilizer formulation; *P = 0.04, Student's t test). The underlying mechanism for these differences is unclear and need further systematic evaluation. However, our results suggest that the choice of oil, solubilizer, and gelling agent are critical to determining the drug release from oleogels. Drug release depends on a complex interplay of the ingredients used in the formulation.

Stability and biocompatibility of oleogels

Long-term stability of drugs is critical and can be challenging to achieve especially in liquid formulations. For example, the commercial dispersible formulation of azithromycin (Zithromax for oral suspension) is supposed to be used within 10 days of suspension and must be stored between 5° and 30°C (*43*). We analyzed the stability of azithromycin in an oleogel formulation at 40°C for a period of >6 weeks. As oxidation can be a major degradation mechanism in oils, we supplemented our formulation with an antioxidant, propyl gallate. Propyl gallate has been commonly used in the food and drug industry at this level and is known to be a good stabilizer in vegetable-based oils (*44*, *45*). Over the period of the study, we did not observe any significant degradation (% azithromycin stable on day 3 was 99.6 ± 8.1, n = 5 versus % azithromycin stable on day 44 was 98.7 ± 5.1, n = 5; P = 0.8, Student's t test) (fig. S4). These results provide initial proof of concept that our gels can be handled in the absence of special storage conditions, thus putting low burden on resources.

Next, we conducted an acute biocompatibility study to ensure that exposure to drug-loaded oleogels is not toxic. We postulated that infants who have difficulty swallowing soft gels may be effectively treated via the rectal route. Hence, the oleogels were administered via both the oral and rectal routes. Tissue biopsies of the stomach and rectum were collected before and 24 hours after treatment with the azithromycin oleogel (fig. S5). Immunohistochemistry analysis of tissue cross sections revealed no toxicity as assessed by a board-certified pathologist.

Pharmacokinetics of oleogels in a large animal model

We characterized the pharmacokinetics of the oleogel and tablet formulations of azithromycin, praziquantel, and lumefantrine in a swine model. The tablet was administered orally as is commonly practiced. The oleogel was administered orally and rectally, which allowed us to compare the effect of formulation and dosing route on the pharmacokinetics.

The pharmacokinetics of azithromycin tablets, oral oleogels, and rectal oleogels is shown in Fig. 5, A to C. The drugs were rapidly absorbed from all formulations and reached maximal concentrations within 3 to 4 hours. The maximal concentration for the tablet, oral gel, and rectal gel were 334 ± 41 ng/ml, 317 ± 54 ng/ml, and 224 ± 39 ng/ml (n = 4 to 6), respectively. The AUC of orally dosed azithromycin oleogel was nearly three times higher than that of the tablet (2896 ± 545 ng*hour/ml versus 823 ± 112 ng*hour/ml, n = 6; *P < 0.05, one-way ANOVA with post-hoc Bonferroni). The rectal oleogel resulted in a twofold increase in bioavailability compared with the tablet (Fig. 5D).

The pharmacokinetics of praziquantel is shown in Fig. 5, E to G. Praziquantel was also rapidly absorbed from both the oral and rectal routes. On average, maximal concentrations were observed at 2.1 ± 1.1 hours and 3.7 ± 0.4 hours for the oral and rectal oleogels. The t_{max} of praziquantel tablets was 3.5 ± 0.2 hours. There was no statistically significant difference between the AUCs of the praziquantel oleogels and the oral tablets (Fig. 5H), which have near-complete bioavailability (*46*).

Last, we analyzed the pharmacokinetics of lumefantrine for oral and rectal administration (Fig. 5, I to K). Regardless of formulation and route of administration, lumefantrine showed a characteristically prolonged half-life. The bioavailability of the oral oleogel was comparable to that of the commercial tablet (Fig. 5L). The AUC observed with the rectal oleogel was about 1⁄16 of that obtained with the oral oleogel (3389 ± 2617 ng*hour/ml versus 55,654 ± 10,912 ng*hour/ml, n = 3 to 5; *P < 0.05, Student's t test). We believe that poor partitioning of the drug from the rectal formulation resulted in lower drug absorption. On the other hand, the high solubility of lumefantrine, a weak base, in gastric acid resulted in complete release of the drug from the oral formulation and a high drug uptake.

Formulation and characterization of oleopastes

Oil-based gels provided unique opportunities for pediatric drug administration, and we showed its utility for three water-insoluble drugs. We were now interested in determining whether drugs with relatively higher water solubility could be formulated in these oil-based systems. To understand this better, we chose to formulate moxifloxacin hydrochloride, a broad-spectrum antibiotic with a water solubility of >10 mg/ml (*47*). As the drug is suspended in these formulations, we referred to them as oleopastes. We evaluated the oleopastes on the basis of three performance parameters—homogeneity, stability, and pharmacokinetics.

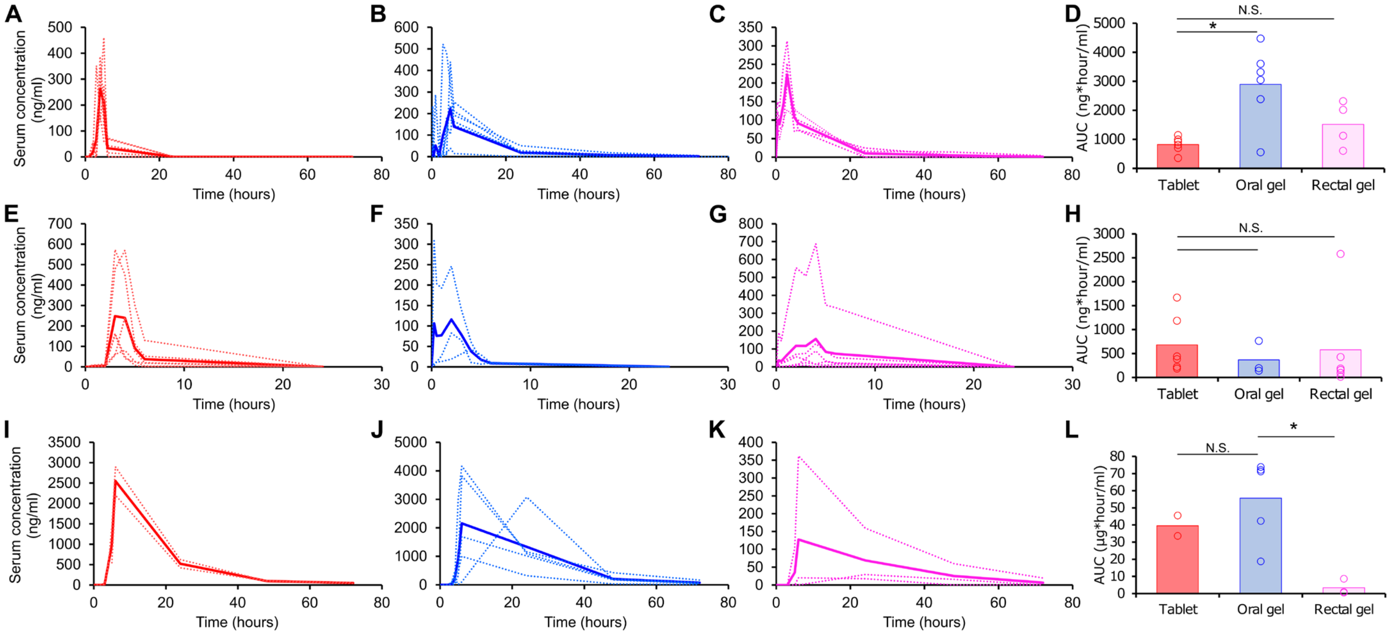

Fig. 5. Pharmacokinetics of oral and rectal oleogels in swine model. Concentration-time profiles of azithromycin in pigs dosed with (**A**) oral tablet, (**B**) oral oleogel, and (**C**) rectal oleogel are shown. The AUC of the three formulations is shown in (**D**). Concentration-time profiles of praziquantel in pigs dosed with (**E**) oral tablet, (**F**) oral oleogel, and (**G**) rectal oleogel are shown. The AUC of the three formulations is shown in (**H**). Concentration-time profiles of lumefantrine in pigs dosed with (**I**) oral tablet, (**J**) oral oleogel, and (**K**) rectal oleogel are shown. The AUC of the three formulations is shown in (**L**). For all pharmacokinetic curves, dotted lines indicate pharmacokinetics in individual animals, and hard lines show average pharmacokinetics. For all AUC assessments, open circles are individual data points, and bars indicate average. *$P < 0.05$; N.S. indicates that differences are not statistically significant; one-way ANOVA with post-hoc Bonferroni.

First, we measured the homogeneity of the suspended drug in the oleopaste. Specifically, oleopastes were stored at 4°C, and the concentration of drug in the top and bottom halves of the oleopaste was evaluated over a month (Fig. 6A). On day 1, concentrations of moxifloxacin hydrochloride in the top and bottom halves of the oleopaste were comparable (191 ± 11 mg/g, $n = 3$ versus 183 ± 10 mg/g, $n = 3$; not significant, one-way ANOVA with post-hoc Bonferroni). On day 30, the differences in concentrations of the drug in the top and bottom halves of the oleopaste were not statistically different (197 ± 14 mg/g, $n = 3$ versus 169 ± 2 mg/g, $n = 3$; not significant, one-way ANOVA with post-hoc Bonferroni). There was no difference in drug concentrations between day 1 and day 30, suggesting that the drug remained stable during this time at 4°C (191 ± 11 mg/g, $n = 3$ versus 197 ± 14 mg/g, $n = 3$; not significant, one-way ANOVA with post-hoc Bonferroni).

In the absence of refrigeration, temperatures can fluctuate during storage and during transport. Hence, we evaluated the homogeneity and short-term stability of oleopastes at 40°C (Fig. 6B). Similar to our results at cold temperatures, drug homogeneity remained intact after 30 days of storing at 40°C [162 ± 12 mg/g (top half), $n = 3$ versus 172 ± 8 mg/g (bottom half), $n = 3$; not significant, one-way ANOVA]. Moreover, there was no statistical difference in drug concentrations found on day 1 and day 30 (166 ± 2 mg/g, $n = 3$ versus 162 ± 12 mg/g, $n = 3$; not significant, one-way ANOVA). Short-term temperature excursions up to 60°C can occur during transport (*48*), and we were interested in understanding whether the oleopastes could tolerate these conditions (Fig. 6C). To evaluate this, we stored the oleopaste at 4° and 60°C for a week before assessing homogeneity and stability. After storing at 60°C for a week, there was no difference between drug concentrations in the top and bottom halves of the oleopaste (194 ± 9 mg/g, $n = 3$ versus 201 ± 7 mg/g, $n = 3$; not significant, one-way ANOVA). There were no differences in drug concentrations between oleopastes stored at 4° and 60°C (207 ± 19 mg/g, $n = 3$ versus 194 ± 9 mg/g, $n = 3$; not significant, one-way ANOVA).

In sum, these data suggested that moxifloxacin hydrochloride could be homogeneously suspended in oil-based formulations. The drug remained evenly suspended in the formulation for up to 30 days at 4° and 40°C and for up to 7 days at 60°C. In addition, our results indicate that moxifloxacin hydrochloride remained stable for the duration of the experiment. These results indicate that redispersing/mixing may not be required before administration. This puts minimal responsibilities on the providers in the field, which makes these systems amenable for mass drug administration campaigns.

Last, we tested the pharmacokinetics of moxifloxacin hydrochloride oleopastes in a swine model and compared it to an aqueous solution of the drug. The pharmacokinetics of the aqueous solution (Fig. 6D) and oleopaste (Fig. 6E) were highly comparable. The average maximal concentration achieved with the aqueous and oily formulations were 1808 ± 530 ng/ml, $n = 3$ and 1914 ± 303 ng/ml, $n = 3$, respectively. Maximal concentrations were achieved 4 to 5 hours following dosing. The average AUCs for the two formulations were 23,191 ± 4717 ng*hour/ml, $n = 3$ (for aqueous solution) and 24,165 ± 1910 ng*hour/ml, $n = 3$ (for oleopaste) ($P = 0.9$, Student's t test) (Fig. 6F). It is worth noting that, while we could achieve a drug solubility of ~10 mg/ml (1% loading) in the aqueous formulation, drug loading in the oleopaste was 20% (w/w). This allowed us to administer the drug dose in a markedly smaller volume of formulation. These data indicate that a water-soluble drug, moxifloxacin hydrochloride, could be successfully formulated and delivered using the oleopaste platform.

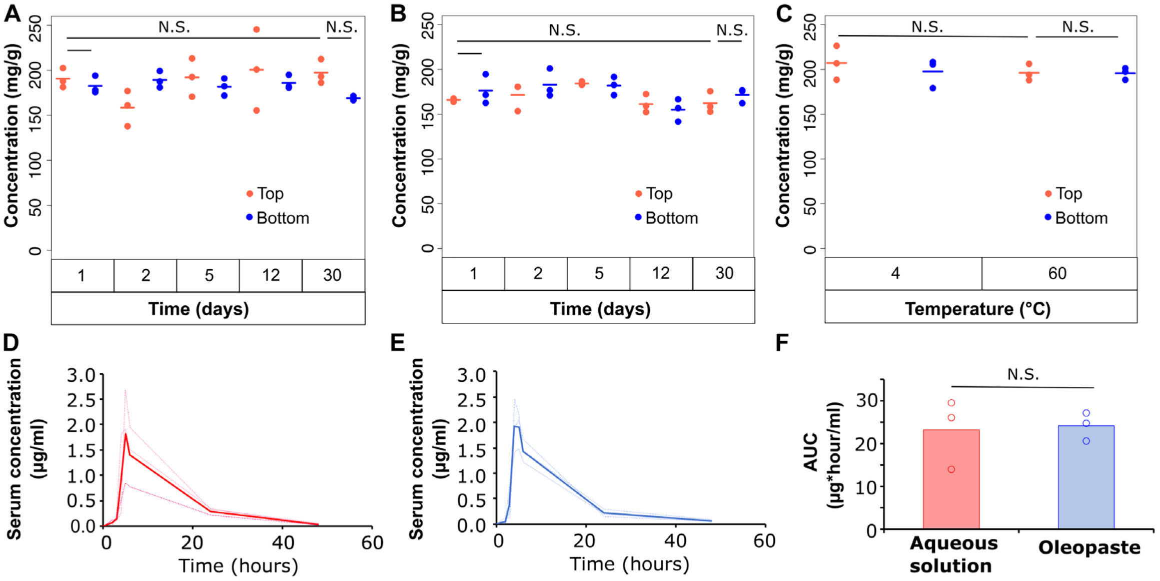

Fig. 6. In vitro and in vivo characterization of moxifloxacin oleopastes. Moxifloxacin oleopastes were synthesized and stored at (**A**) 4°C or (**B**) 40°C. Drug concentrations in the top and bottom halves of the pastes were measured at various time points. Closed circles represent individual data points, and horizontal lines indicate the average value. One-way ANOVA with post-hoc Bonferroni. (**C**) Moxifloxacin oleopastes were stored at 4° or 60°C for 1 week, following which drug concentrations in the top and bottom halves of the paste were measured. Open circles represent individual data points, and horizontal lines indicate the average value. One-way ANOVA with post-hoc Bonferroni. Moxifloxacin was administered orally to swine as an (**D**) aqueous solution or (**E**) oleopaste, and systemic drug concentrations were measured. Dotted lines indicate pharmacokinetics in individual animals, and hard lines show average pharmacokinetics ($n = 3$). (**F**) AUC for animals treated with moxifloxacin aqueous solution and oleopaste. Closed circles are individual data points, and bars indicate average. Student's *t* test.

Sensory evaluation of oleogels

After obtaining approval from our institutional review board, we enlisted a panel of trained tasters to evaluate the sensory attributes of the oleogels—namely, texture and flavor. The intensity of the sensory attributes for each sample was assessed using the Spectrum Descriptive Analysis Method. The analysis was conducted in two stages, with data interpreted to inform selections for subsequent evaluations in the context of a pediatric population. In the first stage, we analyzed oils using flavor descriptive analysis and selected oils that imparted more neutral to favorable flavors (i.e., oils with low flavor intensity, little to no off-notes, and low bitterness, which are likely to be associated with higher compliance). In the second stage of testing, gelling agents were added to the oils to form oleogels, and a two-phase analysis was conducted. First, we screened oleogels to select those with favorable flavors and textures. We applied similar criteria as in the first stage for flavor. For texture, we selected oleogels that created a texture experience close to that of existing pediatric applications. With the selected oleogels, we conducted an in-depth descriptive analysis to yield an in-depth understanding of the flavor and texture of the oleogels. We present results of all three tests here.

In stage 1 of testing, we evaluated the flavor profile of 11 vegetable-based oils. The oils could be categorized into three flavor profiles. First, we identified oils such as cottonseed oil, which did not show a strong signal on any of the taste attributes being analyzed (Fig. 7A). Other examples of such neutral oils included corn oil and safflower oil (table S11). Next, we found oils such as flaxseed oil that showed a higher intensity of bitter flavor. Last, we found that oils such as sesame oil elicit sweeter/nuttier notes (Fig. 7A). Other examples of similar oils included coconut oil and palm oil. Oils with a weaker taste profile or those with sweeter flavors were considered attractive for our applications and were used to make oleogels for the next stage of testing.

In stage 2 of testing, we used five oils (corn, cottonseed, safflower, sesame, and soybean oil) because of their lack of strong flavors or presence of slightly sweet flavors. These oils were combined with three gelling agents (beeswax, carnauba wax, and candelilla wax), which were used at three concentrations. Using these ingredients, we synthesized 45 oleogel formulations (Fig. 7B). Before conducting an in-depth flavor analysis, we performed a screen to select the most promising candidates. In this preliminary screen, we made several key observations regarding the oleogel formulations. First, we identified that the formulations elicited textures that ranged from ones comparable to thickened beverage to ones that resembled a yogurt or pudding (Fig. 7C). Such a range of textures was expected, given the results of our rheology studies showing the differences in viscosities of the oleogels. However, these studies validated that the differences in viscosities in the oleogels were substantial enough to be perceivable by the human palate. In general, we found that oleogels that contained candelilla wax had a bitter taste in the mouth and aftertaste, and these oleogels were considered not appropriate for future studies. As mentioned before, corn oil, cottonseed oil, safflower oil, and soybean oil had a weaker overall flavor, which made these oils attractive for stage 2 of testing. However, we found that, when mixed with the gelling agents, corn oil had too weak of a flavor, and soybean oil had flavors that are less likely to be well accepted by consumers. In contrast, cottonseed oil and safflower oil had a stronger flavor intensity. This detracted from other unfavorable flavors in the formulation. This effect was even more pronounced with sesame oil, and oleogels made with it had a nutty flavor profile. Hence, oleogels containing cottonseed oil, safflower oil, and sesame oil were favored for future studies. We made some observations

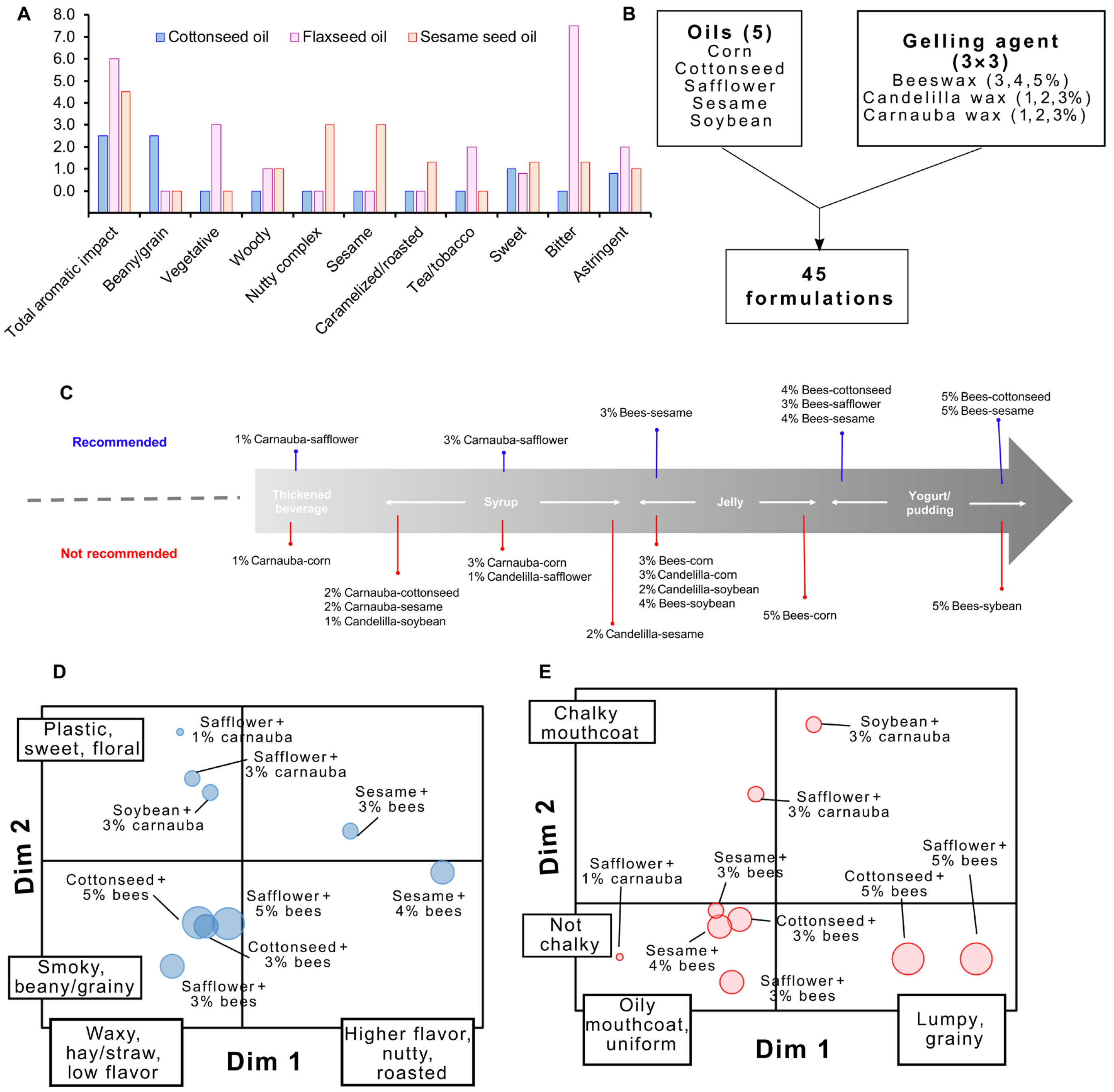

Fig. 7. Sensory evaluation of oleogels. (**A**) Flavor profiles of cottonseed oil, flaxseed oil, and sesame oil. (**B**) Composition of oleogel formulations chosen for stage 2 of testing. (**C**) Texture attributes and favorability of oleogels tested in stage 2. (**D**) Texture map and (**E**) flavor map revealed from in-depth sensory analysis of nine oleogel formulations. Size of the circles in (D) and (E) indicates the thickness of the gel.

regarding the individual formulations, and these are listed in table S12. For example, some formulations (for, e.g., cottonseed oil + 2% carnauba wax and soybean oil + 1% candelilla wax) had a texture of a thin oily syrup. Because such a texture is typically not associated with familiar pediatric products, we removed these formulations from future studies. Several formulations contained a grainy mouthfeel (like Cream of Wheat instant cereal); however, safflower oil with 1% carnauba wax lacked such a texture. As a result, this formulation was considered for in-depth analysis. On the basis of this information, we chose nine formulations for in-depth flavor and texture characterization.

The results of our in-depth characterization are shown in Fig. 7 (D and E). The formulations represent a range of flavor and texture characteristics and can be classified into three groups. First, formulations with midrange levels of carnauba wax (3%) were associated with a higher chalky feel in the mouth and after expectoration. This

chalky texture is typically not associated with familiar pediatric products and is not recommended for further development. Another group of formulations includes those with sesame seed oil. They had a slight grainy and chalky feel and higher overall flavor and higher nutty flavor, with slightly higher bitterness in the formulation with more beeswax (4%). Most of the formulations fall in the third group, with low moderate flavor intensity and low grainy and/or chalky texture. Among this group, safflower oil + 1% carnauba wax had a smoother texture, reminiscent of a thickened beverage with slight chalkiness, and it was lower impact with sweet flavor. Safflower oil and cottonseed oil combined with beeswax yielded formulations that were midrange in flavor impact with the presence of hay/straw and smoky aromatics.

Across the range of textures evaluated, formulations with 5% beeswax had lumpy character, reminiscent of cottage cheese in addition to grainy and chalky feel in the mouth. Lower beeswax formulations were less rough during manipulation and had higher mixes with saliva. Beeswax formulations lack the higher chalky texture detected in 3% carnauba wax formulations. Overall, the nine formulations examined here demonstrate a range of flavor and texture possibilities.

Single- and multidose containers for dispensing oleogels

We set out to understand the ideal packaging that would enable easy dispensing and metered dosing of the oleogels and oleopaste. Two applicator designs were tested—plastic ampoules or unit dose packaging (commercially procured from LF of America) and multidose applicators designed and fabricated in-house (Fig. 8A). We tested two characteristics: the filling uniformity of the applicators and the dispensing reproducibility of the formulation from the applicators.

We hand-filled oleogels in 12 single-dose containers and gravimetrically determined the filling consistency. On average, we filled 2.85 g of the oleogel into each container with an SD of 4.5%. We then asked three volunteers to dispense gels from the containers. Overall, the three individuals were able to dispense 64.7 ± 5.4% (n = 12) of the gel. The SDs for the three individuals dispensing the gels were 2.5% (volunteer 1; n = 4), 6.5% (volunteer 2; n = 4), and 6.3% (volunteer 3; n = 4) (Fig. 8B).

Our multidose containers were designed to have four pockets each, with each pocket sealed off from the next. We were able to fill the container with 10.6 ± 0.3 g (n = 9) of gel. Three volunteers were able to dispense 90.8 ± 5% (n = 9) of the formulation from the container, markedly higher than what was dispensed from the single-dose container. For the three individuals, dispensing across pockets was highly consistent; the average doses dispensed from the four pockets were 2.4 ± 0.2 g (pocket 1; n = 9), 2.4 ± 0.1 g (pocket 2; n = 9), 2.4 ± 0.3 g (pocket 3; n = 9), and 2.4 ± 0.3 g (pocket 4; n = 9) (Fig. 8C). Last, there was a high consistency of quantities dispersed across the three individuals: 9.7 ± 0.4 g (volunteer 1; n = 3), 9.3 ± 0.6 g (volunteer 2; n = 3), and 9.9 ± 0.4 g (volunteer 3; n = 3) (Fig. 8D). These data indicate a proof of principle for packaging and dispensing of the oleogel/oleopaste formulations.

DISCUSSION

Administering medicines to children in resource-limited settings has unique challenges. First, body weights among children can vary

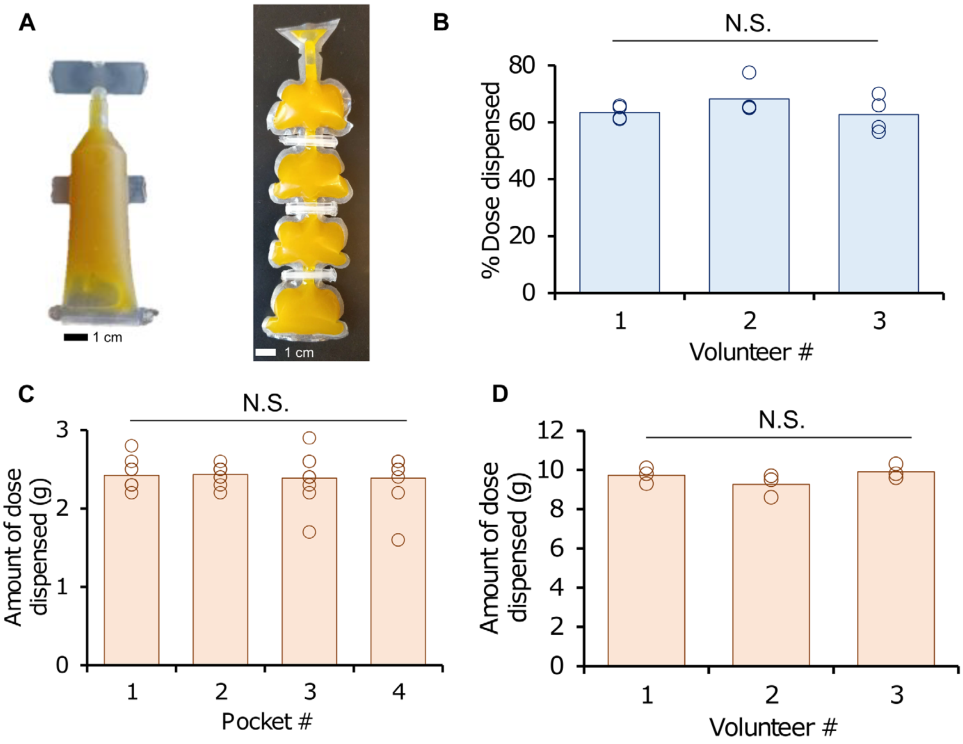

Fig. 8. Macrofluidic device for dosing of oleogels and oleopastes. (**A**) Images of single- and multidose devices for dispensing oleogels and oleopastes. (**B**) Quantification of dose dispensed from a single-dose device across three volunteers. (**C**) Measurement of dose dispensed from four pockets of the multidose dispenser. (**D**) Comparison of dose dispensed from a multidose dispenser across three volunteers. Open circles are individual data points, and bars indicate the average. One-way ANOVA.

substantially, which requires allometric scaling of drug doses. Children may not be able to swallow solid dosage forms, and most dosage forms are available as tablets. This requires trained pharmacists/nurses/medical doctors for on-field reformulation, and such trained personnel may not always be available. Because of lack of cold supply chains, the drug product may be exposed to extreme weather conditions. Last, budgets for pediatric medicines may be restrained, warranting cheap products. In this study, we describe the development of an oleogel platform to overcome these challenges. We show that the oleogel formulation can be made with safe ingredients, remain stable for extended times at high temperatures, are capable of delivering drugs at levels comparable to or better than commercial tablets, can be used for drugs with a range of physicochemical properties, and can be administered using a metered dosage form with favorable sensory feedback maximizing the likelihood of patient acceptability. Hence, we believe that our system overcomes a central challenge in global health.

The formulations described here—oleogels—are inspired by the manipulation of oils described in the food industry. The choice of oleogels as candidate drug carriers was motivated by four factors. First, as most drugs are hydrophobic, oils can serve as an excellent solvent. Second, oils have a long-standing history of use in people and hence have an established safety profile. Third, the manufacturing of oleogels is very simple and scalable, involving only three-unit operations viz. heating, mixing, and cooling. Last, oleogels are amenable to be dosed by both the oral and rectal routes, with the former not involving swallowing of a hard solid. Hence, these have the potential to be used in newborns, infants, and children. The purpose of this study was to attempt to develop drug formulations that could be easily administered to children.

Oleogels were composed of three inactive ingredients, namely, gelling agents, solubilizers, and oils. Here, we analyzed the effect of each of these ingredients on the physicochemical properties of the oleogels. We showed that the choice of gelling agent could affect the viscosity of the formulation, as well as the melting temperature of the formulation. Gelling agents with higher melting temperatures are desired to produce oleogels with high heat stability (*16*, *18*). However, formulation synthesis would need to occur at a higher temperature when using gelling agents with higher melting points. This may not be suitable for all drugs. Hence, the gelling agent must be selected carefully to balance long-term physical stability of the formulation with heat stability of the drug during synthesis.

We also focused on understanding the effect of oil and solubilizer on drug solubility. Expectedly, we observed that the addition of solubilizers had a tremendous effect on the solubility of the drug in the oleogel base. However, note that drug solubility in the oil-solubilizer mixture was not a mere arithmetic sum of its solubility in the individual components. Solubilizers were able to enhance drug solubility in some oils better than in others. The mechanism for this phenomenon was not studied here but might be of interest in future studies. It should be noted that we used the solubilizers at different concentrations, which were determined by the maximum levels that they have been used before (*49*). This was done to obtain translationally relevant information. However, future studies analyzing the effect of drug solubility in the same concentration of various solubilizers may be of academic interest.

A notable finding of this report is that oleogels and oleopastes may perform similarly to or better than commercial tablets. We show that this system is highly versatile and can be used for the delivery of a variety of drugs. The four drugs tested in this paper—azithromycin, praziquantel, lumefantrine, and moxifloxacin—had distinct water solubilities and octanol-water partition coefficients (fig. S6). However, it is important to note that these formulations are effective only if used via the correct route of administration. Hence, although they are a promising base for drug delivery, oleogels will likely need to be optimized for each drug of interest.

There are several questions that currently remain unanswered. In our pharmacokinetic studies, we chose the formulation in which the drug had the highest solubility. However, it is unclear whether drug solubility in the formulation ensures that the drug does not precipitate in physiological fluid and whether drug solubility in the formulation is the only determinant of drug absorption. Not all drugs dissolved in our formulation base, and further optimization may be required for delivery of drugs that are insoluble in our formulation base. To widen the scope of these formulations, introduction of excipients that enhance drug solubility in physiological fluid and particle engineering techniques may be warranted. However, our data suggest that water-soluble drugs that are not soluble in oily formulations may be delivered well with this technique. Last, much is known about the digestion of oily excipients and their influence on drug absorption (*50*). Here, we have not probed the mechanisms of drug release from our formulations and whether formulating drugs in oleogels affects the pathway of absorption. A more in-depth characterization may be required to address these questions.

In summary, we describe here gels made from food-based oils with highly malleable physicochemical properties that can be used for delivering drugs to children. We show the basic properties of this drug delivery platform designed for a highly vulnerable patient population and believe that these formulations will be an important tool toward improving overall health and well-being in children.

METHODS

Materials

Oils, palmitic acid, 12-hydroxystearic acid, behenic acid, lauric acid, arachidic acid, 12-hydroxylauric acid, linolenic acid, 16-hydroxypalmitic acid, 2-hydroxycaprioic acid, stearyl alcohol, stearyl methacrylate, stearyl amine, oleic acid, stearic acid, glyceryl monooleate [90% United States Pharmacopeia (USP) reference standard], L-α-phosphatidylcholine [from egg yolk; type XVI-E, ≥99% (TLC), lyophilized powder], maleic acid (99% pure), sodium taurocholate hydrate, sodium oleate, α-amylase from human saliva (type IX-A, lyophilized powder; 1000 to 3000 U/mg of protein), pepsin from porcine gastric mucosa (3200 to 4500 U/mg of protein), pancreatin (8×USP specifications), and 4-bromophenylboronic acid (4-BBBA, ≥95.0%) were purchased from Sigma-Aldrich. 3-Hydroxymyristic acid was purchased from Tokyo Chemical Industry. Linolenic acid, linoelaidic acid, and elaidic acid were purchased from Cayman Chemical Company. Rice bran wax and castor oil wax were purchased from HalalEveryday. Carnauba wax was purchased from Luxuriant. Beeswax was purchased from Stakich Inc. Candelilla wax was purchased from Plant Guru Inc. Soy wax was obtained from Golden Brands. Lauroglycol FCC, Labrafil M1944, Labrafil M2125, Labrasol ALF, Plurol oleique, Lauroglycol 90, Labrafac lipophile, Maisine, Capryol PGMC, Peceol, and Capryol 90 were a gift from Gattefossé (Saint Priest, France).

Inversion assay to measure gelling capacity of gelling agents

The gelling agents were mixed with corn oil in increasingly higher concentrations, heated to 5° to 10°C above their melting point, and then cooled to room temperature. Gel formation was determined using a vial inversion test. Combinations that did not flow to the bottom of the vial were considered gels.

Rheology

Rheological analysis for the gels was performed on a TA AR2000 rheometer equipped with a 60-mm 2° cone upper geometry with a Peltier stage. The gel mixtures were heated past their melting point and then transferred to the Peltier stage, which was heated to the same temperature. The upper cone was lowered to ensure complete contact with the gel, and the stage was then cooled to 25°C to allow the melted mixture to congeal. Excess gel was removed using a spatula. Dynamic oscillatory strain amplitude sweep measurements were performed at a frequency of 1 Hz to estimate the viscoelastic region for the gels. After determining the linear viscoelastic region, dynamic oscillatory frequency sweep measurements were performed at 0.01% strain amplitude. For these studies, storage modulus (G') was used as a measure of gel strength. All the measurements were analyzed using the TA Universal analysis software.

Bright-field microscopy

The microstructure of the gels with various concentrations of gelling agents was examined with light microscopy. A drop of each molten gel sample was deposited on a heated glass slide and slightly pressed with a coverslip to ensure the formation of a thin film. Samples were allowed to cool at ambient temperature for 24 hours before being visualized using a Nikon Eclipse ME600 light microscope (Nikon Instruments Inc., NY, USA). Images were acquired with a DS-Ri1 camera (Nikon Instruments Inc., NY, USA).

Differential scanning calorimetry

The thermal behavior (melting and crystallization) of the gel was evaluated using DSC (DSC-8000, PerkinElmer, USA) equipped with an Intracooler 2 cooling accessory. Data were analyzed using Pyris (version 11.0.0.0449, PerkinElmer). Samples in the molten state were weighed in aluminum pans (ca. 5 mg), hermetically sealed, and left at ambient temperature for 24 hours before analysis. Specimens were subjected to three heating/cooling cycles from 20° to 120°C at a rate of 4°C/min and from 120° to 20°C at the same rate under a nitrogen purge of 20 ml/min.

Measuring drug solubility in oil-solubilizer mixtures

We measured the solubility of three anti-infectives—azithromycin, praziquantel, and lumefantrine—in 108 formulations prepared by mixing various oils and solubilizers (9 oils × 11 solubilizers, and 9 oils without solubilizer). Given our interest in rapid clinical translation, we limited the weight fraction of the solubilizer to the maximum level that they were used in FDA-approved products. The concentration of the solubilizers used in our studies is shown in table S1.

We measured approximately 2 g of the oil-solubilizer mixtures in a 20-ml glass vial. The drug was added in excess to each formulation, and the mixture was stirred overnight at room temperature. One milliliter of the mixture was removed, placed in a microcentrifuge tube, and centrifuged at 6000 rpm for 15 min to remove insoluble drug particles. A fraction of the supernatant was removed, and the drug was extracted using methanol or acetonitrile. Drug concentrations in the extracts were measured using high-performance liquid chromatography (HPLC).

An Agilent 1260 Infinity II HPLC system was equipped with a quaternary pump, autosampler, thermostat, control module, and diode array detector. Data processing and analysis were performed using OpenLab CDS ChemStation. Praziquantel chromatographic separations were carried out on an Agilent ZORBAX Eclipse Plus C-18 analytical column (4.6 mm by 150 mm) with 5-μm particles, maintained at 40°C. Gradient separation at a flow rate of 1 ml/min was achieved using water and acetonitrile, which corresponds to A and B, respectively. The run time was 5 min with a 3-min post-run and the following gradient profile: [0 min, A: 70%, B: 30%] and [2.5 min, A: 30%, B: 70%]. The injection volume was 5 μl. The diode array detector was set using an ultraviolet (UV) detection wavelength of 217 nm with no reference at an acquisition rate of 40 Hz. This method is summarized in table S2.

Azithromycin chromatographic separations were carried out on an Agilent 4.6-mm by 150-mm ZORBAX Eclipse XDB C-18 analytical column with 5-μm particles, maintained at 50°C. Isocratic separation at a flow rate of 0.75 ml/min was achieved using 20 mM ammonium acetate in water:acetonitrile:methanol at a volume ratio of 20:20:60. The run time was 7 min. The injection volume was 5 μl. The diode array detector was set using a UV detection wavelength of 210 nm with no reference at an acquisition rate of 5 Hz. Agilent 6120B Single Quadrupole hardware connected with a liquid nitrogen tank was operated at 100°C and in selective ion monitoring mode. The system was set up at 350°C of gas temperature, 35 psi of nebulizer pressure, and 10 liter/min of drying gas flow rate. This method is summarized in table S3.

Lumefantrine chromatographic separations were carried out on an Agilent Poroshell 120 PFP column (4.6 mm by 100 mm) with 2.7-μm particles, maintained at 40°C. Gradient separation at a flow rate of 1 ml/min was achieved using 0.1% formic acid in water and methanol, which corresponds to A and B, respectively. The run time was 7 min with a 3-min post-run and a gradient profile of the following: 0 min, A: 30% and B: 70%; 3 min, A: 5% and B: 95%. The injection volume was 10 μl. The diode array detector was set using a UV detection wavelength of 303 nm with no reference wavelength at an acquisition rate of 40 Hz. This method is summarized in table S4.

Synthesis of drug-loaded oleogels

To synthesize the oleogels, oil and solubilizer were accurately weighed and briefly vortexed to ensure mixing. To this mixture, drug was added, and the suspension was placed in a bath sonicator for about 1 hour to dissolve the drug. The drug solution was then placed on a hot plate and heated to a temperature of 5° to 10°C above the melting point of the gelling agent. The gelling agent was added to the formulation while stirring. Once the gelling agent melted (5 to 10 min), the molten solution was removed from the hot plate and placed at room temperature to cause gel formation. If needed, the mixture was filled into syringes or other container of choice while molten and then allowed to gel in situ. Unless specified otherwise, the formulations used in these studies are listed in tables S6 to S8.

Analysis of stability of azithromycin oleogels

Azithromycin oleogel was prepared as described above with one change. In that, propyl gallate [0.1% (w/w)] was added to the oil and

solubilizer before addition of the drug. The oleogels were stored at 40°C. At different times, ~1 g of the oleogel was aliquoted. Azithromycin from the aliquots was extracted with 10 ml of methanol for 24 hours. On the next day, the samples were rigorously mixed and transferred to microcentrifuge tubes. The tubes were centrifuged (3220*g*, 10 min) to remove the undissolved gel components of the gel. The supernatant was diluted 1000 times and analyzed using LC–mass spectroscopy (LC-MS).

In vitro digestion and drug bioaccessibility studies

We characterized the digestion of gels in three simulated conditions—oral, gastric, and intestinal phases (fasted state).

Oral conditions

SSF (pH 7.0) was prepared in the presence of α-amylase based on a previously described method (*23*, *51*) with slight modifications. Briefly, SSF stock solution (1.25× concentrated; 3.5 ml) was placed in a glass vial. The 1.25× SSF stock solution contained the following ingredients: 15.1 mM potassium chloride, 3.7 mM potassium dihydrogen phosphate, 13.6 mM sodium bicarbonate, 0.15 mM magnesium chloride, and 0.06 mM ammonium carbonate. To this, 0.5 ml of α-amylase solution (0.3 mg/ml in SSF stock solution), 0.025 ml of 0.3 M calcium chloride, and 0.975 ml of water were added and thoroughly mixed.

The SSF was added to 0.5 g of praziquantel-loaded gel placed in a preheated glass vial at 37°C containing five glass beads to aid in mixing. The vials were placed in an incubator shaker and shaken at 160 rpm and 37°C. One hundred microliters of the sample was withdrawn periodically and centrifuged at 14,000 rpm for 15 min. The supernatant was collected and syringe-filtered through a 0.22-μm nylon syringe filter (Nalgene Syringe Filters, Thermo Fisher Scientific). Drug concentration in the filtrate was analyzed using HPLC.

Gastric conditions

In vitro digestion in the gastric phase was performed in 7.5 ml of SGF (pH 2) [sodium chloride (2 g/liter) and concentrated hydrochloric acid (2.917 g/liter)] in the presence of pepsin (3.4 g/liter). SGF was added to 0.5 g of gel placed in a preheated glass vial at 37°C containing five glass beads to aid in mixing. The vials were shaken as in the oral condition. Samples (0.2 ml) were withdrawn periodically, centrifuged at 14,000 rpm for 15 min, and syringe-filtered through 0.22-μm nylon syringe filters (Nalgene Syringe Filters, Thermo Fisher Scientific) before drug quantification with HPLC.

Intestinal phase in fasted conditions

The fasting state simulated intestinal fluid version 2 (FaSSIF-V2) medium (pH 6.5) was prepared on the basis of a previously described method (*52*) in the presence of pancreatin (8×USP specification). To initiate digestion, 50 ml of FaSSIF-V2 medium was added in 0.1 g of gel placed in a preheated glass vial at 37°C containing five glass beads to aid mixing. Periodically, samples (0.4 ml) were withdrawn and immediately inhibited with the addition of 3 μl of 1 M 4-BBBA before ultracentrifugation in polycarbonate tubes for 60 min at 40,000 rpm and 37°C (ultracentrifuge OptimaTM TL with rotor type TLA 120.1, Beckman Coulter, CA, USA). One hundred microliters was carefully withdrawn from the aqueous micellar phase and diluted with 700 μl of acetonitrile. Samples were then centrifuged for 15 min at 14,000 rpm at room temperature, and drug concentration in the supernatant was measured using HPLC.

Cryo-TEM of the in vitro lipolysis under FaSSIF conditions

Visualization of the lipolytic products under fasted state conditions was performed with cryo-TEM. In vitro digestion of the gel was initiated in FaSSIF as described above. Samples were withdrawn at 30, 60, and 120 min; inhibited with the addition of 4-BBBA; and immediately processed for cryo-TEM. Three microliters of each sample was deposited on a lacey copper grid coated with a continuous carbon film and blotted to remove excess sample without damaging the carbon layer (Gatan Cryoplunge III). The grid was mounted on a Gatan 626 single-tilt cryo-holder in the TEM column. The specimen and holder tip were cooled down with liquid nitrogen, which was maintained during transfer into the microscope and subsequent imaging. The images were recorded with a charge-coupled device camera (Gatan 2kx2k UltraScan) on a JEOL 2100 FEG microscope under low-dose conditions that were essential to avoid sample damage under the electron beam. The microscope was operated at 200 kV and with a magnification in the ranges of 10,000 to 60,000 for assessing particle size and distribution.

Effect of formulation components on the bioaccessibility of azithromycin

We determined the effect of varying formulation components on the bioaccessibility of azithromycin. To perform these studies, we chose three oils (cottonseed oil, corn oil, and soybean oil) and three gelling agents (beeswax, carnauba wax, and candelilla wax). Formulations contained Capryol 90, Peceol, or Maisine as the solubilizer or were made devoid of solubilizer. This yielded three oil conditions × three gelling agent conditions × four solubilizer conditions = 36 formulations. The composition of the drug, solubilizer, gelling agent, and oil are listed in table S9. To synthesize these gels, oil and solubilizer were mixed in a 20-ml glass vial. To this, the mixture drug was added and dissolved by sonication in a water bath. A magnetic stir bar was added to the drug solution, and the mixture was placed on a hot plate at 90°C. The gelling agent was added to this mixture. Upon melting of the gelling agent, the molten solution was removed from the hot plate and placed at room temperature to enable gel formation.

Drug release was measured in simulated intestinal fluid, and drug concentration was measured using LC-MS as described above. Here, we report individual drug release curves. To perform a head-to-head comparison, we calculated the AUC of each of the release curves using the trapezoidal rule. Mean values of the AUC are also reported.

Synthesis and characterization of moxifloxacin oleopastes

Moxifloxacin oleopastes were synthesized by a process similar to the synthesis of the oleogels, but with some modifications. Oil was weighed in a 20-ml glass vial, and moxifloxacin hydrochloride was added to it. The mixture was sonicated in a water bath to ensure complete dispersion of the drug. The mixture was then placed on a hot plate at a temperature of 5° to 10°C above the melting point of the gelling agent. The gelling agent was added to the suspension and stirred for 10 min. The hot suspension was then drawn into syringes and transferred to the container of choice. The container was cooled by placing into an ice bath, which led to the formation of the paste. The formulation components and concentrations are listed in table S10.

We assessed the stability and homogeneity of moxifloxacin in the oleopaste over a month-long interval. For these studies, the moxifloxacin oleopaste was placed in glass vials that were stored at 4° or 40°C for a month. At different times, glass vials were removed from their respective storage conditions and allowed to equilibrate to room temperature. Three aliquots from the top and bottom halves of the oleopaste were weighed. Drug was extracted overnight

from these aliquots using methanol. Following extraction, the methanol phase was separated by centrifugation at 10,000 rpm for 10 min. Drug concentration in the methanol extract was measured using a Tecan plate reader or using HPLC. For HPLC analysis, an Agilent Poroshell EC C18 (4.6 mm by 50 mm; 2.7 μm) maintained at 50°C was used as the stationary phase. The aqueous and organic portions of the mobile phase were 0.1% phosphoric acid in water and acetonitrile, respectively. A gradient method was used to achieve separation: 0 min, A: 95% and B: 5%; 2.5 min, A: 5% and B: 95%. Solvents were flown at 1 ml/min. The injection volume was 5 μl. The total run time was 5 min with a post-run time of 3 min. Absorbance was measured at 303 nm. This method is summarized in table S5.

We also measured drug stability and homogeneity at 60°C. These studies were performed in a way identical to the stability studies at 4° and 40°C. However, it was performed for a shorter duration (1 week). Drug was extracted using methanol, and concentrations were measured using HPLC.

Oral and rectal pharmacokinetics in pigs

All procedures conformed to the protocols approved by the Massachusetts Institute of Technology Committee on Animal Care. The pharmacokinetics of azithromycin, praziquantel, lumefantrine, and moxifloxacin were characterized in a large animal model. Female Yorkshire pigs weighing approximately 30 to 75 kg were placed on liquid diet 24 hours before the experiment and fasted overnight with access to water ad libitum. Animals were sedated with intramuscular injection of Telazol (tiletamine/zolazepam) (5 mg/kg), xylazine (2 mg/kg), and atropine (0.04 mg/kg), and after intubation, anesthesia was maintained with isoflurane (1 to 3% isoflurane + 2 to 3% oxygen inhaled). A central venous catheter was then inserted using the Seldinger technique to allow for frequent blood sampling. The oleogel was directly administered endoscopically to the stomach using a catheter placed down the working channel of the endoscope. The catheter was used to prevent loss of any oleogel inside the working channel of the endoscope. A 60-ml syringe was then used to push the oleogel through the tubing and into the stomach. The administered drug dose was 5 mg/kg for azithromycin, 20 mg/kg for praziquantel, 24 mg/kg for lumefantrine, and 5 mg/kg for moxifloxacin. The formulations of azithromycin, praziquantel, and lumefantrine gels are shown in tables S6 to S8 and S10. Commercial tablets of azithromycin, praziquantel, and lumefantrine were administered in gelatin capsules via an oral gavage tube with 200 ml of water using an oral syringe. Moxifloxacin was administered as an aqueous solution at a concentration of 10 mg/ml. Rectal gels were dosed with a syringe, inserted ~10 cm inside the anal cavity to ensure administration in the rectum. Blood samples were collected in serum separator tubes and centrifuged at 3202*g* for 10 min at 4°C. Serum was separated and stored at −80°C until LC–tandem MS (LC-MS/MS) analysis.

LC-MS/MS analysis of drug concentrations in serum

Stock solutions of each compound was prepared in methanol at a concentration of 500 μg/ml. A 12-point calibration curve was prepared in analyte-free, blank serum ranging from 1.25 to 5000 ng/ml. One hundred microliters of each serum sample was spiked with 200 μl of internal standard in acetonitrile (250 ng/ml) to elicit protein precipitation. Samples were vortexed, sonicated for 10 min, and centrifuged for 10 min at 13,000 rpm. Two hundred microliters of supernatant was pipetted into a 96-well plate containing 200 μl of water. Analyte concentrations in serum were quantified using ultrahigh-performance LC-MS/MS (UPLC-MS/MS). Analysis was performed on a Waters ACQUITY UPLC I-Class System aligned with a Waters Xevo TQ-S mass spectrometer (Waters Corporation, Milford, MA). Liquid chromatographic separation was performed on an Acquity UPLC BEH C18 (50 mm by 2.1 mm; 1.7-μm particle size) column for praziquantel, azithromycin, and lumefantrine or an Acquity UPLC CSH C18 (50 mm by 2.1 mm; 1.7-μm particle size) column for moxifloxacin at 50°C. The mobile phase consisted of aqueous 0.1% formic acid and 10 mM ammonium formate solution (mobile phase A) and acetonitrile: 10 mM ammonium formate and 0.1% formic acid solution [95:5 (v/v)] (mobile phase B). The mobile phase had a continuous flow rate of 0.6 ml/min using a time and solvent gradient composition.

For the analysis of praziquantel, the initial composition, 80% mobile phase A, was held for 0.50 min, following which the composition was changed linearly to 0% mobile phase A over the next 2.00 min. The composition of 0% mobile phase A and 100% mobile phase B was held constant until 3.50 min. The composition returned to 80% mobile phase A at 3.51 min and was held at this composition until the completion of the run, ending at 5.00 min, where it remained for column equilibration. The total run time was 5.00 min.

For the analysis of azithromycin, the initial composition, 100% mobile phase A, was held for 1.00 min. Following which, the composition was changed linearly to 50% mobile phase A and 50% mobile phase B until 1.25 min. At 1.50 min, the composition was 20% mobile phase A. At 2.50 min, the composition was 100% mobile phase B, where it was held constant until 3.00 min. At 3.25 min, the composition returned to 100% mobile phase A, where it remained for column equilibration for the duration of the run, ending at 4.00 min.

For the analysis of lumefantrine, the initial composition of 70% mobile phase A was held until 0.50 min. The concentration was changed linearly to 0% mobile phase A and 100% mobile phase B until 2.50 min, where it was held until 3.50 min. At 3.51 min, the composition returned to 70% mobile phase A, where it remained for column equilibration for the remainder of the run. The total run time was 5.00 min.

For the analysis of moxifloxacin, the initial composition of 100% mobile phase A was held until 1.00 min. The concentration was changed linearly to 50% mobile phase A and 50% mobile phase B over the next 0.25 min. At 1.50 min, the composition was 20% mobile phase A, and at 2.50 min, the composition was 0% mobile phase A and 100% mobile phase B, which was held constant until 3.00 min. The composition returned to 100% mobile phase A at 3.25 min and was held at this composition until completion of the run, ending at 4.00 min, where it remained for column equilibration.

Last, 1.00, 10.00, 2.00, and 4.00 μl were injected onto the UPLC-ESI-MS system for analysis of praziquantel, azithromycin, lumefantrine, and moxifloxacin, respectively. Sample introduction and ionization was performed by electrospray ionization (ESI) in the positive ionization mode. Waters MassLynx 4.1 software was used for data acquisition and analysis.

The mass-to-charge transitions [mass/charge ratio (m/z)] used to quantitate praziquantel were 313.22 > 203.09 and 313.22 > 83.01 for quantitation and confirmation, respectively. For internal standard, mebendazole, 296.06 > 264.03 and 296.06 > 76.99 m/z transitions were used for quantitation and confirmation, respectively.

The mass-to-charge transitions (m/z) used to quantitate azithromycin were 749.732 > 116.087 and 749.732 > 83.06 for quantitation and

confirmation, respectively. For internal standard, roxithromycin, 837.81 > 158.14 and 837.81 > 116.09 *m*/*z* transitions were used for quantitation and confirmation, respectively.

The mass-to-charge transitions (*m*/*z*) used to quantitate lumefantrine were 528.28 > 346.06 and 528.28 > 276.21 for quantitation and confirmation, respectively. For internal standard, artemisinin, 283.234 > 247.17 and 283.234 > 125.135 *m*/*z* transitions were used for quantitation and confirmation, respectively.

The mass to charge transitions (*m*/*z)* used to quantitate moxifloxacin were 402.21 > 110.12 and 402.21 > 358.21 for quantitation and confirmation, respectively. For internal standard, ciprofloxacin, 332.10 > 231.09 and 332.10 > 245.14 *m*/*z* transitions were used for quantitation and confirmation, respectively.

We present the serum concentration-time profiles for each animal, as well as the average pharmacokinetic profile. To compare the bioavailabilities of the formulations, we calculated the AUC using the trapezoidal rule.

Sensory evaluation of oleogels

Procedures for sensory evaluation of the oleogels conformed to the protocols approved by the Massachusetts Institute of Technology Institutional Review Board/Committee on the Use of Humans as Experimental Subjects.

Formulation of oleogels

The vegetable oils were placed in a beaker on a hot plate stirrer. The oils were heated to a temperature above the melting temperature of the gelling agent. Specifically, to make oleogels containing beeswax, candelilla wax, and carnauba wax, the oils were heated to 75°, 82°, and 92°C, respectively. A fixed amount of gelling agent was added to oil while stirring. The gelling agent melted almost instantaneously. The beaker was removed from the hot plate, the stir bar was retrieved, and the mixture was transferred to an open-mouth mason jar and allowed to cool to room temperature to yield the gel. The gels were stored in a refrigerator overnight before testing.

Test sessions

All members of the panel convened in the presence of the project manager for simultaneous test sessions. Six to 12 panelists were present for each session. Each panel member interested in participating was allowed to participate only if they read and agreed to sign an informed consent form before participating in the first test session. Each panelist received one ~88 ml sample to evaluate flavor and texture. Panel members followed a sip and spit procedure to expectorate all samples after evaluation. Panelists recorded individual ratings using a customized ballot, and then each attribute was discussed until a consensus value is decided upon and recorded by the panel leader. This evaluation process was be followed repeatedly on scheduled days until all samples had been evaluated.

Study design

This study design involved monadic assessments by a trained panel of oleogel formulations to develop a flavor and texture profile. The intensity of the sensory attributes for each sample was assessed using the Spectrum descriptive analysis method.

Test methodology—Quantitative descriptive analysis

Sensory Spectrum panelists are trained using the Spectrum descriptive analysis method. Panelists are trained on a universal scale that focuses on the intensity or strength of the signal coupled with detailed description and definitions of sensory attributes. Panelists are selected on their ability to detect and discriminate differences in aromatics, basic tastes, and texture properties. Panelists train extensively through a series of lectures, courses, and ongoing project-specific training with experienced panelists. The Spectrum descriptive analysis method grounds itself in the use of published intensity reference scales to define intensity boundaries in sensory experiences. Panelists are also exposed to a wide variety of qualitative attribute references to clarify and refine the definition of each chosen attribute. Panelists receive hundreds of hours of training and ongoing performance feedback with respect to use of the scale and attribute language.

Use of a universal scale allows attributes to be compared in intensity to one another, e.g., comparing the intensity of sweet taste to the intensity of bitter taste. The use of a universal scale with defined intensity references also allows for comparison of samples within and across studies and products.

Sensory Spectrum's panel rates the intensity of all attributes on a 15-point intensity scale with 0 = none and 15 = very strong. The intensity scale uses 0.1-point increments for a possible 151 points of differentiation. Panelists are trained to use the scale and to use the scale in a similar way across panelists and across samples. For this study, consensus data will be collected and analyzed. Sensory attributes and intensities are objective, technical data generated by the panelists. Data are interpreted by sensory scientists as favorable and unfavorable, and recommendations are made in the context of a pediatric population.

Manufacturing of multidose dispenser

To manufacture the multidose applicator, 7.62 cm thin-film polyethylene (PE) sleeves (McMaster Carr) were used as the base packaging material. A 2D vector design of the applicator was prepared in Adobe Illustrator for laser etching using a 60-W CO_2 laser cutter (Universal Laser Systems). The design features 4- to 3-ml doses of pods that are daisy-chained or connected together in a linear fashion with a notched opening at the top for the application (Fig. 6A). Methods of sealing and resealing were explored, including clamping or twisting the channel that separated the pods. The proof of concept was tested using a hermetic zipper, also known as a zip lock seal. The plastic sleeves were secured down to acrylic sheets using tape, and air was evacuated before sealing the opening of the sleeves. The sleeves were etched using 10P 90S etching settings and then removed, and excess material was trimmed to form the blank multidose applicators. To load the multidose applicators, oleogels prepared in syringes were secured to the opening of the applicator with zip ties and then dispensed into the applicator until full. The openings of the applicators were sealed with a tabletop impulse sealer (McMaster Carr).

The plastic ampoules or unit dose packaging featured a blow-molded vessel with a one-time use twist-off cap. The unit dose applicators were filled in a similar manner to the multidose packaging; 3 ml of the oleogel was injected into the packaging and then sealed with the tabletop impulse sealer. Both the applicators and syringe were weighed before loading and after loading.

Both packaging forms were tested to understand usability and reproducibility. Three volunteers received four unit dose applicators and three multidose applicators (with four doses total) and were instructed to dispense each dose into a weighing boat. The weighing boats were weighed between each dose, and participants were surveyed to understand usability. From the study, participants were able to extract 90.8% of the viscous oleogel from the multidose applicator while extracting 64.8% of the gel from the unit dose applicator. From surveying the participants, they stated that the

thin-film multidose applicator did not require as much force to squeeze out the oleogel as compared to the unit dose applicator; however, the multidose applicator was complex to use. Participants had difficulty extracting the dosages through the linear channels when using the multidose applicators. When considering the final packaging form of the oleogels, we would consider combining both approaches—thin-film packaging and unit dose packaging in series. Thin-film packaging does not require much force to extract the viscous oleogels, and packaging in series can allow users to tear off the required dosage for a single administration.

SUPPLEMENTARY MATERIALS

Supplementary material for this article is available at https://science.org/doi/10.1126/sciadv.abm8478

View/request a protocol for this paper from *Bio-protocol*.

REFERENCES AND NOTES

1. GBD 2016 Mortality Collaborators, Global, regional, and national under-5 mortality, adult mortality, age-specific mortality, and life expectancy, 1970–2016: A systematic analysis for the Global Burden of Disease Study 2016. *Lancet* **390**, 1084–1150 (2017).
2. L. Liu, S. Oza, D. Hogan, Y. Chu, J. Perin, J. Zhu, J. E. Lawn, S. Cousens, C. Mathers, R. E. Black, Global, regional, and national causes of under-5 mortality in 2000–15: An updated systematic analysis with implications for the Sustainable Development Goals. *Lancet* **388**, 3027–3035 (2016).
3. N. Golding, R. Burstein, J. Longbottom, A. J. Browne, N. Fullman, A. Osgood-Zimmerman, L. Earl, S. Bhatt, E. Cameron, D. C. Casey, L. Dwyer-Lindgren, T. H. Farag, A. D. Flaxman, M. S. Fraser, P. W. Gething, H. S. Gibson, N. Graetz, L. K. Krause, X. R. Kulikoff, S. S. Lim, B. Mappin, C. Morozoff, R. C. Reiner, A. Sligar, D. L. Smith, H. Wang, D. J. Weiss, C. J. L. Murray, C. L. Moyes, S. I. Hay, Mapping under-5 and neonatal mortality in Africa, 2000–15: A baseline analysis for the Sustainable Development Goals. *Lancet* **390**, 2171–2182 (2017).
4. J. D. Keenan, R. L. Bailey, S. K. West, A. M. Arzika, J. Hart, J. Weaver, K. Kalua, Z. Mrango, K. J. Ray, C. Cook, E. Lebas, K. S. O'Brien, P. M. Emerson, T. C. Porco, T. M. Lietman; MORDOR Study Group, Azithromycin to reduce childhood mortality in sub-saharan Africa. *N. Engl. J. Med.* **378**, 1583–1592 (2018).
5. J. Polaha, W. T. Dalton III, B. M. Lancaster, Parental report of medication acceptance among youth: Implications for everyday practice. *South. Med. J.* **101**, 1106–1112 (2008).
6. J. W. Kernell, R. V. De Paola, A. M. Maglione, L. N. Ahern, N. G. Penney, D. G. Addiss, Risk of adverse swallowing events and choking during deworming for preschool-aged children. *PLOS Negl. Trop. Dis.* 12, e0006578 (2018).
7. World Health Organization, Action against worms (2007); https://www.who.int/publications/i/item/WHO-HTM-NTD-PCT-2007.3.
8. H. Zhang, D. Wang, R. Butler, N. L. Campbell, J. Long, B. Tan, D. J. Duncalf, A. J. Foster, A. Hopkinson, D. Taylor, D. Angus, A. I. Cooper, S. P. Rannard, Formation and enhanced biocidal activity of water-dispersable organic nanoparticles. *Nat. Nanotechnol.* **3**, 506–511 (2008).
9. M. Giardiello, N. J. Liptrott, T. O. McDonald, D. Moss, M. Siccardi, P. Martin, D. Smith, R. Gurjar, S. P. Rannard, A. Owen, Accelerated oral nanomedicine discovery from miniaturized screening to clinical production exemplified by paediatric HIV nanotherapies. *Nat. Commun.* **7**, 13184 (2016).
10. M. Siccardi, P. Martin, D. Smith, P. Curley, T. McDonald, M. Giardiello, N. Liptrott, S. Rannard, A. Owen, Towards a rational design of solid drug nanoparticles with optimised pharmacological properties. *J. Interdiscip. Nanomedicine* **1**, 110–123 (2016).
11. U.S. Food and Drug Administration, Over-the-counter drug products intended for oral ingestion that contain alcohol (1993); www.accessdata.fda.gov/scripts/cdrh/cfdocs/cfcfr/CFRSearch.cfm?CFRPart=328&showFR=1.
12. E. Marek, W. K. Kraft, Ethanol pharmacokinetics in neonates and infants. *Curr. Ther. Res.* **76**, 90–97 (2014).
13. R. H. Richey, U. U. Shah, M. Peak, J. V. Craig, J. L. Ford, C. E. Barker, A. J. Nunn, M. A. Turner, Manipulation of drugs to achieve the required dose is intrinsic to paediatric practice but is not supported by guidelines or evidence. *BMC Pediatr.* **13**, 81 (2013).
14. A. Zajicek, M. J. Fossler, J. S. Barrett, J. H. Worthington, R. Ternik, G. Charkoftaki, S. Lum, J. Breitkreutz, M. Baltezor, P. Macheras, M. Khan, S. Agharkar, D. D. MacLaren, A report from the pediatric formulations task force: Perspectives on the state of child-friendly oral dosage forms. *AAPS J.* **15**, 1072–1081 (2013).
15. R. B. Shah, J. S. Collier, V. A. Sayeed, A. Bryant, M. J. Habib, M. A. Khan, Tablet splitting of a narrow therapeutic index drug: A case with levothyroxine sodium. *AAPS PharmSciTech* **11**, 1359–1367 (2010).
16. M. A. Rogers, T. Strober, A. Bot, J. F. Toro-Vazquez, T. Stortz, A. G. Marangoni, Edible oleogels in molecular gastronomy. *Int. J. Gastron. Food Sci.* **2**, 22–31 (2014).
17. E. D. Co, A. G. Marangoni, Organogels: An alternative edible oil-structuring method. *J. Am. Oil Chem. Soc.* **89**, 749–780 (2012).
18. T. A. Stortz, A. G. Marangoni, Heat resistant chocolate. *Trends Food Sci. Technol.* **22**, 201–214 (2011).
19. A. R. Patel, N. Cludts, M. D. Bin Sintang, A. Lesaffer, K. Dewettinck, Edible oleogels based on water soluble food polymers: Preparation, characterization and potential application. *Food Funct.* **5**, 2833–2841 (2014).
20. K. Colla, A. Costanzo, S. Gamlath, Fat replacers in baked food products. *Foods* **7**, 192 (2018).
21. K. Iwanaga, T. Sumizawa, M. Miyazaki, M. Kakemi, Characterization of organogel as a novel oral controlled release formulation for lipophilic compounds. *Int. J. Pharm.* **388**, 123–128 (2010).
22. F. R. Lupi, D. Gabriele, N. Baldino, P. Mijovic, O. I. Parisi, F. Puoci, Olive oil/policosanol organogels for nutraceutical and drug delivery purposes. *Food Funct.* **4**, 1512–1520 (2013).
23. C. M. O'Sullivan, M. Davidovich-Pinhas, A. J. Wright, S. Barbut, A. G. Marangoni, Ethylcellulose oleogels for lipophilic bioactive delivery—Effect of oleogelation on in vitro bioaccessibility and stability of beta-carotene. *Food Funct.* **8**, 1438–1451 (2017).
24. C. M. O'Sullivan, S. Barbut, A. G. Marangoni, Edible oleogels for the oral delivery of lipid soluble molecules: Composition and structural design considerations. *Trends Food Sci. Technol.* **57**, 59–73 (2016).
25. World Health Organization, WHO model list of essential medicines for children (2017); https://apps.who.int/iris/bitstream/handle/10665/273825/EMLc-6-eng.pdf?ua=1.
26. A. Puşcaş, V. Mureşan, C. Socaciu, S. Muste, Oleogels in food: A review of current and potential applications. *Foods* **9**, 70 (2020).
27. A. M. Nel, S. C. Smythe, S. Habibi, P. E. Kaptur, J. W. Romano, Pharmacokinetics of 2 dapivirine vaginal microbicide gels and their safety vs. hydroxyethyl cellulose-based universal placebo gel. *J. Acquir. Immune Defic. Syndr.* **55**, 161–169 (2010).
28. A. R. Kirtane, M. K. Rothenberger, A. Frieberg, K. Nephew, N. Schultz-Darken, T. Schmidt, T. Reimann, A. T. Haase, J. Panyam, Evaluation of vaginal drug levels and safety of a locally administered glycerol monolaurate cream in rhesus macaques. *J. Pharm. Sci.* **106**, 1821–1827 (2017).
29. J. L. Stringer, N. A. Peppas, Diffusion of small molecular weight drugs in radiation-crosslinked poly(ethylene oxide) hydrogels. *J. Control. Release* **42**, 195–202 (1996).
30. H. H. Tønnesen, J. Karlsen, Alginate in drug delivery systems. *Drug Dev. Ind. Pharm.* **28**, 621–630 (2002).
31. O. S. Fenton, J. L. Andresen, M. Paolini, R. Langer, β-Aminoacrylate synthetic hydrogels: Easily accessible and operationally simple biomaterials networks. *Angew. Chemie* **130**, 16258–16261 (2018).
32. B. Jeong, Y. H. Bae, D. S. Lee, S. W. Kim, Biodegradable block copolymers as injectable drug-delivery systems. *Nature* **388**, 860–862 (1997).
33. S. Kumar, K. J. Himmelstein, Modification of in situ gelling behavior of carbopol solutions by hydroxypropyl methylcellulose. *J. Pharm. Sci.* **84**, 344–348 (1995).
34. E. A. Appel, M. W. Tibbitt, M. J. Webber, B. A. Mattix, O. Veiseh, R. Langer, Self-assembled hydrogels utilizing polymer–nanoparticle interactions. *Nat. Commun.* **6**, 6295 (2015).
35. D. C. Zulim Botega, A. G. Marangoni, A. K. Smith, H. D. Goff, The potential application of rice bran wax oleogel to replace solid fat and enhance unsaturated fat content in ice cream. *J. Food Sci.* **78**, C1334–C1339 (2013).
36. P. Talens, J. M. Krochta, Plasticizing effects of beeswax and carnauba wax on tensile and water vapor permeability properties of whey protein films. *J. Food Sci.* **70**, E239–E243 (2005).
37. S. Saucedo-Pompa, R. Rojas-Molina, A. F. Aguilera-Carbó, A. Saenz-Galindo, H. de La Garza, D. Jasso-Cantú, C. N. Aguilar, Edible film based on candelilla wax to improve the shelf life and quality of avocado. *Food Res. Int.* **42**, 511–515 (2009).
38. A. I. Blake, E. D. Co, A. G. Marangoni, Structure and physical properties of plant wax crystal networks and their relationship to oil binding capacity. *J. Am. Oil Chem. Soc.* **91**, 885–903 (2014).
39. U.S. Department of Agriculture, FoodData Central (2020).
40. S. Timoumi, D. Mangin, R. Peczalski, F. Zagrouba, J. Andrieu, Stability and thermophysical properties of azithromycin dihydrate. *Arab. J. Chem.* **7**, 189–195 (2014).
41. O. A. Kotila, O. O. Olaniyi, A. O. Adegoke, C. P. Babalola, Experimental determination of the physicochemical properties of lumefantrine. *Afr. J. Med. Med. Sci.* **42**, 209–214 (2013).
42. J. Kuntsche, J. C. Horst, H. Bunjes, Cryogenic transmission electron microscopy (cryo-TEM) for studying the morphology of colloidal drug delivery systems. *Int. J. Pharm.* **417**, 120–137 (2011).
43. Pfizer Labs, FDA label—Zithromax (2013); www.accessdata.fda.gov/drugsatfda_docs/label/2013/050710s039,050711s036,050784s023lbl.pdf.
44. K. C. Waterman, R. C. Adami, K. M. Alsante, J. Hong, M. S. Landis, F. Lombardo, C. J. Roberts, Stabilization of pharmaceuticals to oxidative degradation. *Pharm. Dev. Technol.* **7**, 1–32 (2002).

45. J. Smith, *Food Additive User's Handbook*, J. Smith, Ed. (Springer US, 1991).
46. J. S. McCarthy, T. A. Moore, Drugs for helminths, in *Mandell, Douglas, and Bennett's Principles and Practice of Infectious Diseases*, J. E. Bennett, R. Dolin, M. J. Blaser, Eds. (Elsevier, ed. 8, 2015), pp. 519–527.e3.
47. F. Varanda, M. J. Pratas de Melo, A. I. Caço, R. Dohrn, F. A. Makrydaki, E. Voutsas, D. Tassios, I. M. Marrucho, Solubility of antibiotics in different solvents. 1. Hydrochloride forms of tetracycline, moxifloxacin, and ciprofloxacin. *Ind. Eng. Chem. Res.* **45**, 6368–6374 (2006).
48. T. I. Lucas, R. H. Bishara, R. H. Seevers, A stability program for the distribution of drug products. *Pharm. Technol.* **28**, 68–73 (2004).
49. U.S. Food and Drug Administration, Inactive ingredient search for approved drug products (2020); www.accessdata.fda.gov/scripts/cder/iig/index.cfm.
50. C. J. H. Porter, N. L. Trevaskis, W. N. Charman, Lipids and lipid-based formulations: Optimizing the oral delivery of lipophilic drugs. *Nat. Rev. Drug Discov.* **6**, 231–248 (2007).
51. M. Minekus, M. Alminger, P. Alvito, S. Ballance, T. Bohn, C. Bourlieu, F. Carrière, R. Boutrou, M. Corredig, D. Dupont, C. Dufour, L. Egger, M. Golding, S. Karakaya, B. Kirkhus, S. Le Feunteun, U. Lesmes, A. Macierzanka, A. Mackie, S. Marze, D. J. McClements, O. Ménard, I. Recio, C. N. Santos, R. P. Singh, G. E. Vegarud, M. S. J. Wickham, W. Weitschies, A. Brodkorb, A standardised static in vitro digestion method suitable for food – an international consensus. *Food Funct.* **5**, 1113–1124 (2014).
52. M. Kilic, J. Dressman, A simplified method to screen for in-vivo performance of oral lipid formulations. *J. Pharm. Pharmacol.* **66**, 615–623 (2014).

Acknowledgments: We thank S. Hershenson, N. Bowers, C. Moreton, R. Black, F. Alvarez, S. Kern, L. Wood, and P. Welkhoff for feedback. We are also grateful to members of the Langer and Traverso laboratories for helpful advice. We thank A. Lytton-Jean and D. Yun in the Peterson Nanotechnology Core Facility for the expert technical assistance. **Funding:** This work was supported by grant OPP1179091 from the Bill and Melinda Gates Foundation to R.L. and G.T. and OPP1213817 to Sensory Spectrum. A.R.K. is grateful to the PhRMA Foundation Postdoctoral Fellowship for financial support. C.K. is thankful to the Fulbright scholarship for financial support. The Nanotechnology Materials Core is supported, in part, by funding provided to the Koch Institute from a National Cancer Institute Cancer Center support grant. **Author contributions:** A.R.K., R.L., and G.T. conceived and designed the research. A.R.K., C.K., A.W., D.F., D.T.H.L., S.S., N.V.P., and J.L. contributed toward synthesis and in vitro characterization of the formulations. K.B. and A.K. conducted sensory evaluation of the formulations. T.H. and A.W. designed and characterized the macrofluidic dispensing device. K.H. and A.L. conducted HPLC and LC-MS/MS analyses for the in vitro and in vivo characterization of the oleogels/oleopastes. A.R.K., C.K., A.W., D.F., A.H., J.C., S.T., K.I., J.L.P.K., N.V.P., and N.U.R. designed and characterized the oleogels/oleopastes in vivo. J.K.L. evaluated the histology samples. A.R.K., C.K., R.L., and G.T. wrote the initial draft of the paper with input from all authors. All authors have read the paper and approve of it. **Competing interests:** R.L., G.T., A.R.K., A.W., and C.K. are inventors on a patent application related to this work filed by Massachusetts Institute of Technology and the Brigham and Women's Hospital Inc. (no. PCT/US2021/032428, filed on 14 May 2021). Complete details of all relationships for profit and not for profit for G.T. can be found at the following link: www.dropbox.com/sh/szi7vnr4a2ajb56/AABs5N5i0q9AfT1IqIJAE-T5a?dl=0. Complete details for R.L. can be found at the following link: www.dropbox.com/s/yc3xqb5s8s94v7x/Rev%20Langer%20COI.pdf?dl=0. The other authors declare that they have no competing interests. **Data and materials availability:** All data needed to evaluate the conclusions in the paper are present in the paper and/or the Supplementary Materials.

Submitted 15 October 2021
Accepted 13 April 2022
Published 27 May 2022
10.1126/sciadv.abm8478
