# Supplementary material for: Development of oil-based gels as versatile drug delivery systems for pediatric applications
Source: Sci Adv. 2022 May 27;8(21):eabm8478. doi: 10.1126/sciadv.abm8478 (PMC9140966; doi:10.1126/sciadv.abm8478)
Supplement: Supplementary file 2 — Figs. S1 to S6 Tables S1 to S12 [file sciadv.abm8478_sm.pdf]

Science Advances
AAAS

Supplementary Materials for

Development of oil-based gels as versatile drug delivery systems for pediatric applications

Ameya R. Kirtane *et al.*

Corresponding author: Giovanni Traverso, cgt20@mit.edu

Sci. Adv. **8**, eabm8478 (2022)
DOI: 10.1126/sciadv.abm8478

The PDF file includes:

Figs. S1 to S6
Tables S1 to S12
Legends for data S1 to S8

Other Supplementary Material in this manuscript includes the following:

Data S1 to S8

Supplementary table 1: Composition of solubilizers used in solubility study

Solubilizer	% w/w
Lauroglycol FCC	0.25
Lauroglycol 90	0.25
Labrafil M1944	2.94
Labrafac lipophile	4
Labrasol ALF	6.9
Labrafil M2125	7.5
Plurol Oleique	8.25
Maisine	8.6
Peceol	10
Capryol 90	10
Capryol PGMC	10

Supplementary table 2: HPLC conditions for analysis of praziquantel

Parameter	Conditions
Column	Agilent Zorbax Eclipse XDB C18 (4.6x150 mm 5 µm)
Injection volume (µl)	5
Flow Rate (ml/min)	1
Column Temperature (C)	40
Elution	Gradient: A water; B acetonitrile: 0 min 50% A, 50% B; 3 min 30% A, 70% B
Run Time (min)	6
Post Run (min)	2.5
Wavelength	217

Supplementary table 3: HPLC conditions for analysis of azithromycin

Parameter	Conditions
Column	Agilent Zorbax Eclipse XDB C18 (4.6x150 mm 5 µm)
Injection volume (µl)	5
Flow Rate (ml/min)	0.75
Column Temperature (C)	50
Elution	Isocratic: 20% 20 mM ammonium acetate (unadjusted); 20% acetonitrile; 60% methanol
Run Time (min)	7
Post Run (min)	0
Mass/Charge (m/z)	749.6

Supplementary table 4: HPLC conditions for analysis of lumefantrine

Parameter	Conditions
Column	Agilent Poroshell EC C18 (4.6x150 mm 2.7 µm)
Injection volume (µl)	5
Flow Rate (ml/min)	1
Column Temperature (C)	50
Elution	Gradient: A 0.1% formic acid in water; B acetonitrile: 0 min 50% A, 50% B; 5 min 5% A, 95% B
Run Time (min)	10
Post Run (min)	3
Wavelength	293

Supplementary table 5: HPLC conditions for analysis of moxifloxacin

Column	Agilent Poroshell EC C18 (4.6x150 mm 2.7 μm)
Injection volume (μl)	5
Flow Rate (ml/min)	1
Column Temperature (C)	50
Elution	Gradient: A 0.1% phosphoric acid in water; B acetonitrile: 0 min 95% A, 5% B; 2.5 min 5% A, 95% B
Run Time (min)	5
Post Run (min)	2
Wavelength	303

Supplementary table 6: Formulation of oral and rectal oleogels of praziquantel

Ingredient	Quantity (%w/w)
Praziquantel	3.8
Ricebran wax (oral/rectal)	8/6
Capryol 90	10
Flaxseed oil	q.s.

Supplementary table 7: Formulation of oral and rectal oleogels of azithromycin

Ingredient	Quantity (%w/w)
Azithromycin dihydrate	1.78
1-octadecanol (oral/rectal)	8/6
Capryol 90	10
Flaxseed oil	q.s.

Supplementary table 8: Formulation of oral and rectal oleogels of lumefantrine

Ingredient	Quantity (%w/w)
Lumefantrine	3.6
Ricebran wax (oral/rectal)	8/6
Capryol 90	10
Flaxseed oil	q.s.

Supplementary table 9: Formulation of azithromycin oleogels for studying effect of formulation components on bioaccessibility

Ingredient	Quantity (%w/w)
Azithromycin dihydrate	0.4
Gelling agent	5
Solubilizer (if used)	10
Oil	q.s.

Supplementary table 10: Formulation of oral and oleopaste of moxifloxacin

Ingredient	Quantity (%w/w)
Moxifloxacin hydrochloride	20
Ricebran wax	4
Cottonseed oil	q.s.

Supplemental Table 11: Flavor profiles of oils alone

Sample	Cottonseed Oil	Rapeseed Oil	Soybean Oil	Corn Oil	Safflower Seed Oil	Sunflower Oil	Coconut Oil	Palm Oil	Flaxseed Oil	Peanut Oil	Sesame Oil
**AROMATICS**											
Total Aromatic Impact	2.5	2	2.8	3.8	2.8	2.8	4	4.8	6	3	4.5
Beany/Grain	2.5	1.5	1.5	1	1.5	1	0	0	0	1.5	0
Green/Fresh	0	0	0.8	0	0.8	0	0	0	0	0	0
Vegetative	0	0	0	0	0	0	0	1.3	3	0	0
Hay/Straw	0	0	0	0	0	0	0	0	0	0	0
Earthy	0	0	0	0	0	0	0	1	0	0	0
Woody	0	0	0	0	0	0	0	2	1	0	1
Nutty Complex	0	1	0	2	1.5	0	2	0	0	1.8	3
Peanut	0	0	0	0	0	0	0	0	0	0	0
Sesame	0	0	0	1.5	0	0	0	0	0	0	3
Coconut Meat	0	0	0	0	0	0	2	0	0	0	0
Sweet Aromatics Complex	0	0	0	0	0	0	2	0	0	0	0
Lactone	0	0	0	0	0	0	2	0	0	0	0
Other Sweet Aromatics (name)	0	0	0	0	0	0	0	0	0	0	0
Caramelized/Roasted	0	0	0	1	0	0	1	1.3	0	0	1.3
Dark Toast	0	0	0	0	0	0	0	0	0	0	0
Fruity	0	0	0	0	0	0	0	0	0	0	0
Floral	0	0	0	0	0	0	0	0	0	0	0
Butter Impression	0	0	0	0	0	0	0	0	0	0	0
Heated Oil	0	0	0.8	0	0	0	0	0	0	0	0
Plastic/Vinyl	0	0	0	0	0	1.5	0	0	0	0	0
Oxidized Complex	0	0.8	0.8	0	0	0	0	0	0	0	0
Cardboard	0	0	0	0	0	0	0	0	0	0	0
Painty	0	0	0	0	0	0	0	0	0	0	0
Fishy	0	0.8	0	0	0	0	0	0	0	0	0
Phenol	0	0	0	0	0	0	0	0	0	0	0
Burnt	0	0	0	0	0	0	0	0	0	0	0
Metallic	0	0	0	0	0	0	0	0	0	0	0
Waxy/Aldehydic	0	0	0	0	0	0.5	0	0	0	0	0
Soapy	0	0	0	0	0	0	0	0	0	0	0
Animal	0	0	0	0	0	0	0	0	0	0	0
Degraded Protein	0	0	0	0	0	0	0	0	0	0	0
Tea/Tobacco	0	0	0	0	0	0	0	0	2	0	0
Other (name)	0	0	0	0	0	0	0	0	0	0	0
**BASIC TASTES**											
Sweet	1	1	1.3	1.5	1	1	1.8	1.5	0.8	1.3	1.3
Sour	0	0	0	0.5	0	0	0	0.5	0	0	0
Salt	0	0	0	0	0	0	0	0.5	0	0	0
Bitter	0	0	0	0	0	0.5	0.5	1	7.5	0	1.3
**CHEMICAL FEELING FACTORS**											
Astringent	0.8	0.5	0	1	0	0	1	1	2	0	1
Metallic	0	0	0	0	0	0	0	0	0	0	0

Supplemental Table 12: Results of screening analysis of 45 oleogel formulations

Oil	Gelling agent	Concentration of gelling agent (%w/w)	Consistency	Recommend/Not recommended	Notes
Corn oil	Beeswax	3	Very loose jelly	Not recommended	
Corn oil	Beeswax	4	Jelly	Not recommended	Waxy mouthcoat
Corn oil	Beeswax	5	Jelly	Not recommended	Waxy mouthcoat
Corn oil	Candelilla wax	1	Thicker syrup	Not recommended	
Corn oil	Candelilla wax	2	Very loose jelly	Not recommended	
Corn oil	Candelilla wax	3	Very loose jelly	Not recommended	
Corn oil	Carnauba wax	1	Thickened beverage	Not recommended	
Corn oil	Carnauba wax	2	Thicker syrup	Not recommended	
Corn oil	Carnauba wax	3	Thicker syrup	Not recommended	
Cottonseed oil	Beeswax	3	Thin yogurt	Recommended	
Cottonseed oil	Beeswax	4	Thin yogurt	Recommended	
Cottonseed oil	Beeswax	5	Yogurt pudding	Recommended	
Cottonseed oil	Candelilla wax	1	Thin syrup	Not recommended	Not recommended due to atypical texture
Cottonseed oil	Candelilla wax	2	Very loose jelly	Not recommended	
Cottonseed oil	Candelilla wax	3	Jelly	Not recommended	Petrolatum-type
Cottonseed oil	Carnauba wax	1	Thin syrup	Not recommended	Not recommended due to atypical texture
Cottonseed oil	Carnauba wax	2	Thin syrup	Not recommended	Not recommended due to atypical texture
Cottonseed oil	Carnauba wax	3	Thin syrup	Not recommended	Not recommended due to atypical texture
Safflower oil	Beeswax	3	Thin yogurt	Recommended	
Safflower oil	Beeswax	4	Thin yogurt	Recommended	
Safflower oil	Beeswax	5	Yogurt pudding	Recommended	
Safflower oil	Candelilla wax	1	Thicker syrup	Not recommended	
Safflower oil	Candelilla wax	2	Very loose jelly	Not recommended	
Safflower oil	Candelilla wax	3	Very loose jelly	Not recommended	
Safflower oil	Carnauba wax	1	Thickened beverage	Recommended	Recommended due to lack of chalky/grainy flavor
Safflower oil	Carnauba wax	2	Thickened beverage	Recommended	
Safflower oil	Carnauba wax	3	Thicker syrup	Recommended	
Sesame oil	Beeswax	3	Thicker syrup	Recommended	Recommended due to high nutty notes
Sesame oil	Beeswax	4	Thin yogurt	Recommended	Recommended due to high nutty notes
Sesame oil	Beeswax	5	Yogurt pudding	Recommended	Nutty notes accompanied with some chalkiness/graininess
Sesame oil	Candelilla wax	1	Thicker syrup	Not recommended	
Sesame oil	Candelilla wax	2	Thick syrup	Not recommended	
Sesame oil	Candelilla wax	3	Very loose jelly	Not recommended	
Sesame oil	Carnauba wax	1	Thin syrup	Not recommended	
Sesame oil	Carnauba wax	2	Thin syrup	Not recommended	Not recommended due to atypical texture
Sesame oil	Carnauba wax	3	Thin syrup	Not recommended	Not recommended due to atypical texture
Soybean oil	Beeswax	3	Very loose jelly	Not recommended	
Soybean oil	Beeswax	4	Very loose jelly	Not recommended	
Soybean oil	Beeswax	5	Yogurt pudding	Not recommended	
Soybean oil	Candelilla wax	1	Thin syrup	Not recommended	Not recommended due to atypical texture
Soybean oil	Candelilla wax	2	Very loose jelly	Not recommended	
Soybean oil	Candelilla wax	3	Very loose jelly	Not recommended	
Soybean oil	Carnauba wax	1	Thin syrup	Not recommended	Not recommended due to atypical texture
Soybean oil	Carnauba wax	2	Thicker syrup	Not recommended	
Soybean oil	Carnauba wax	3	Thicker syrup	Not recommended	

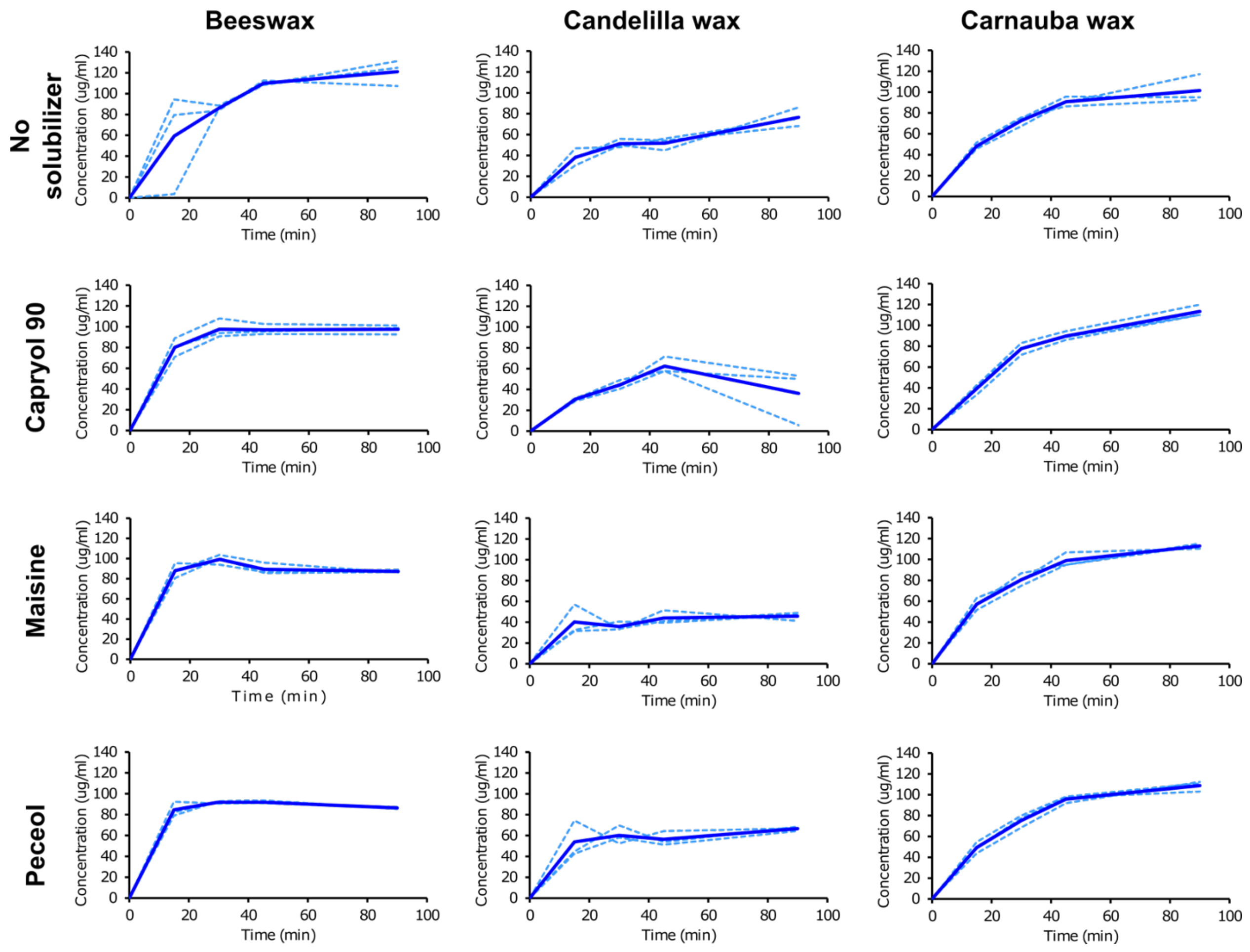

Supplementary Figure 1: Drug release from azithromycin oleogels made using cottonseed oil. Dotted lines indicate individual data, and hard line indicates average.

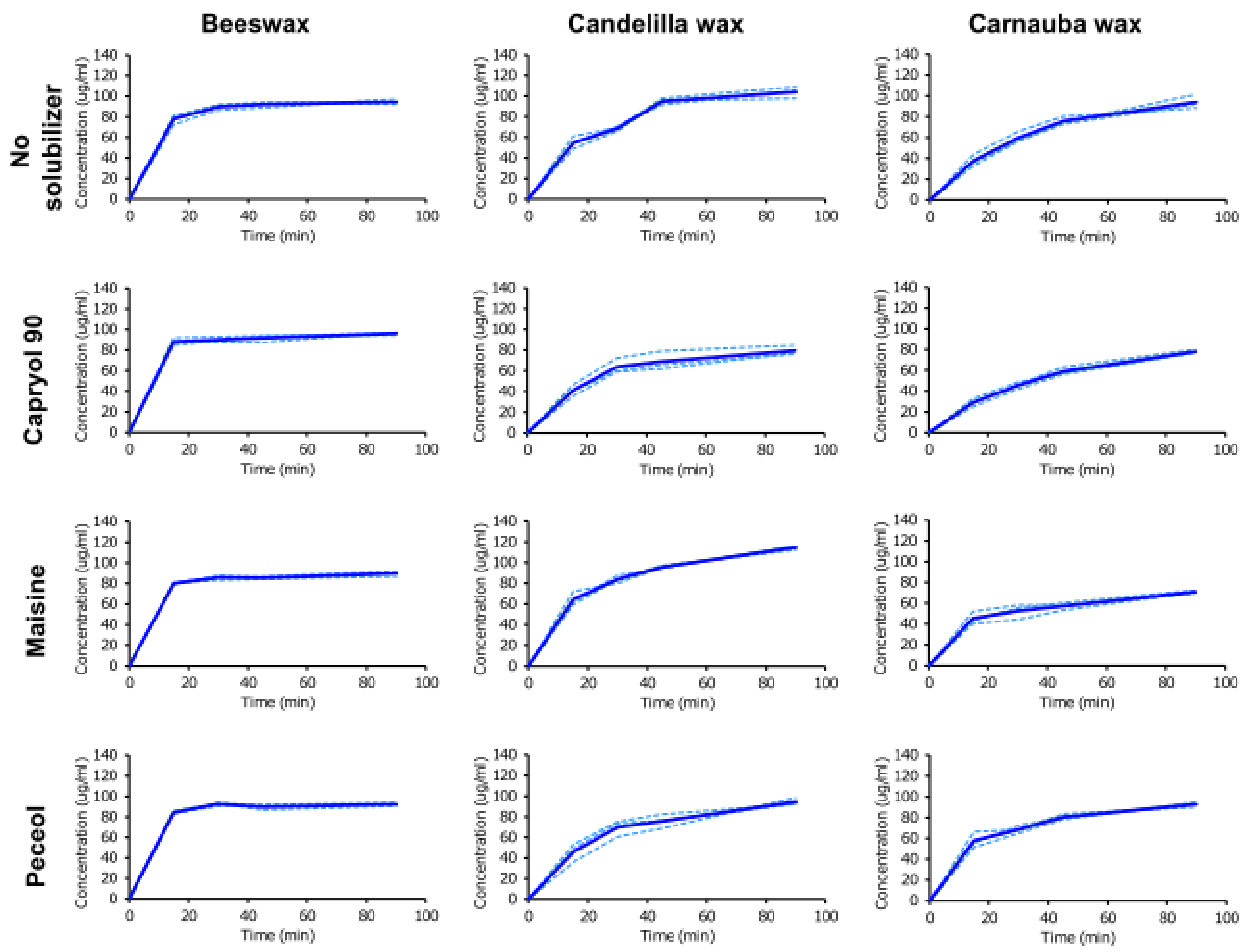

Supplementary Figure 2: Drug release from azithromycin oleogels made using corn oil. Dotted lines indicate individual data, and hard line indicates average.

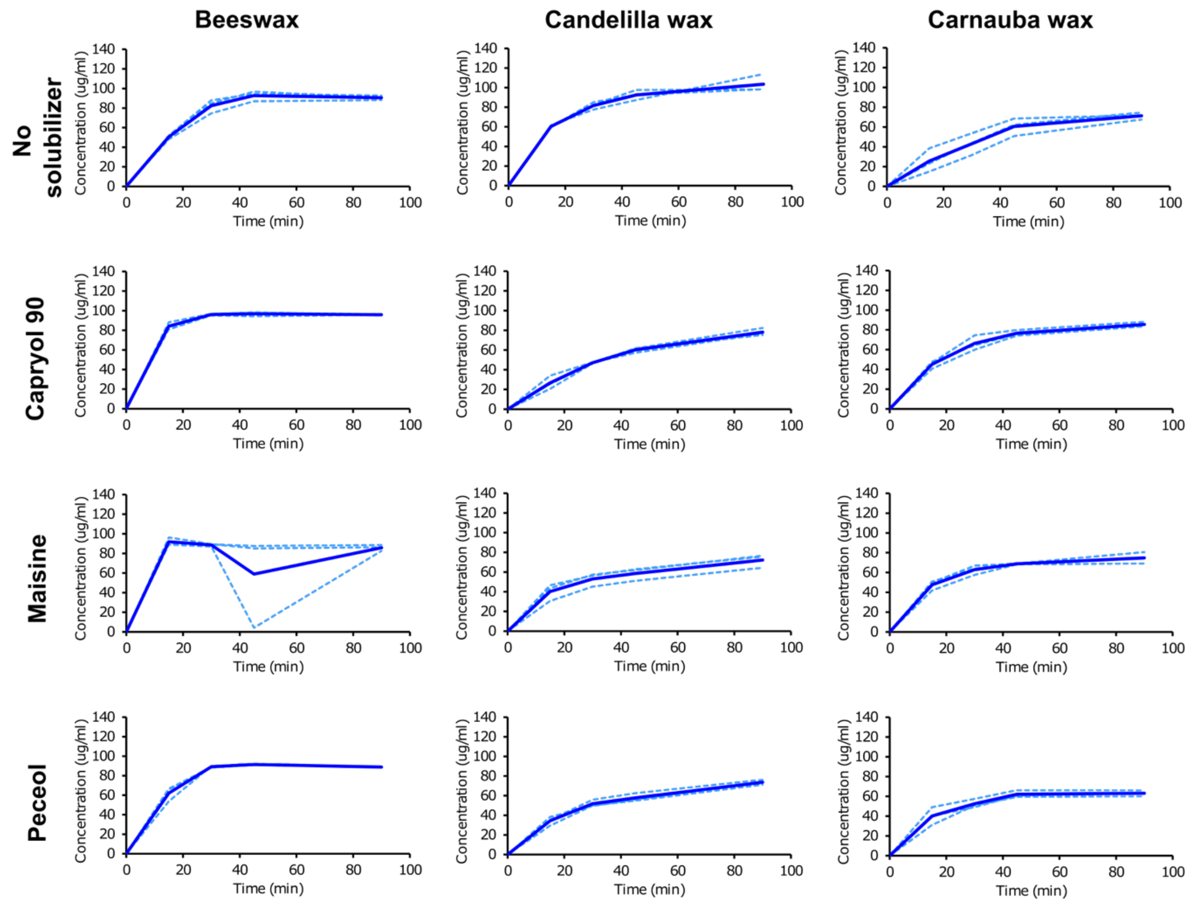

Supplementary Figure 3: Drug release from azithromycin oleogels made using soybean oil. Dotted lines indicate individual data, and hard line indicates average.

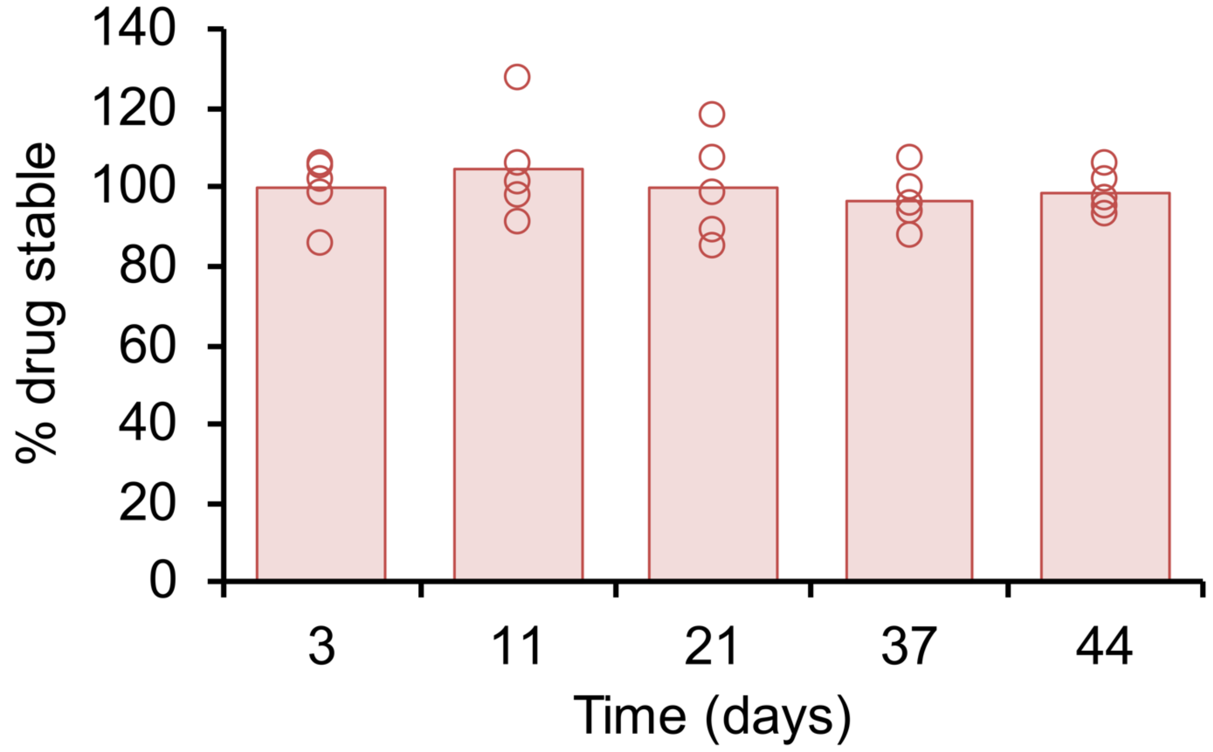

Supplementary figure 4: Stability of azithromycin in oleogel at 40°C.

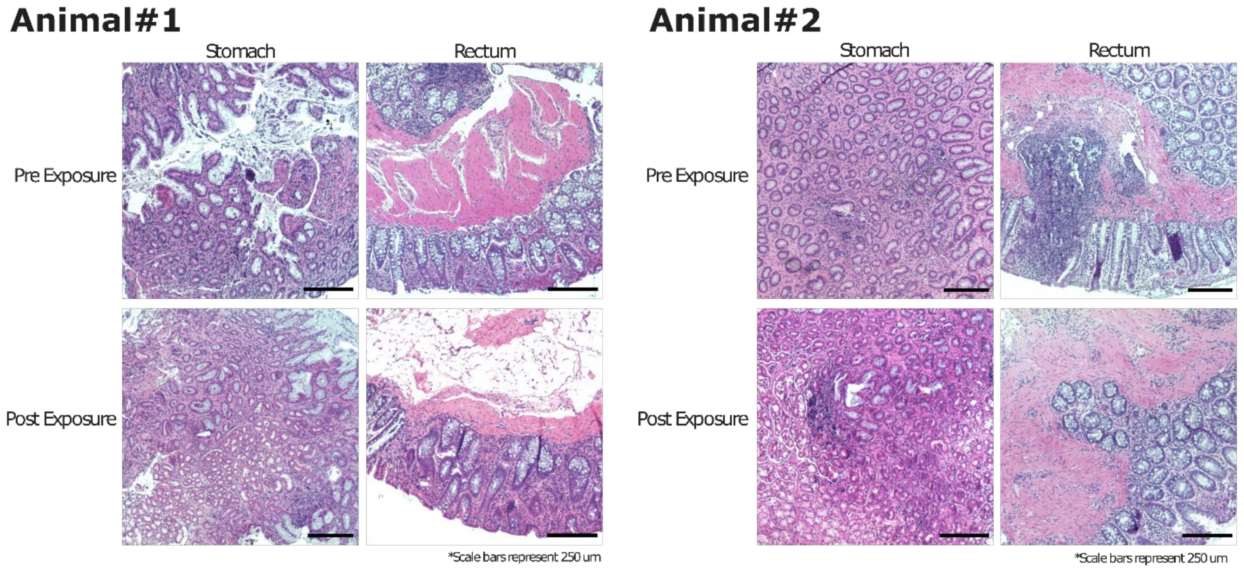

	Pretreatment	Post-treatment
Stomach	**Normal gastric body-/fundic mucosa** *Description:* There is no evidence of parietal cell hyperplasia, activity or chronicity	**Normal gastric antral mucosa** *Description:* Lamina propria accentuated in the luminal compartment. On field shows a small lymphoid follicle. There is no evidence of activity or chronicity.
Rectum	**Normal colorectal mucosa** *Description:* There is no evidence of activity or chronicity.	**Normal colorectal mucosa** *Description:* There is no evidence of activity or chronicity.

Supplementary figure 5: Biocompatibility of azithromycin oleogel upon oral and rectal administration in pigs.

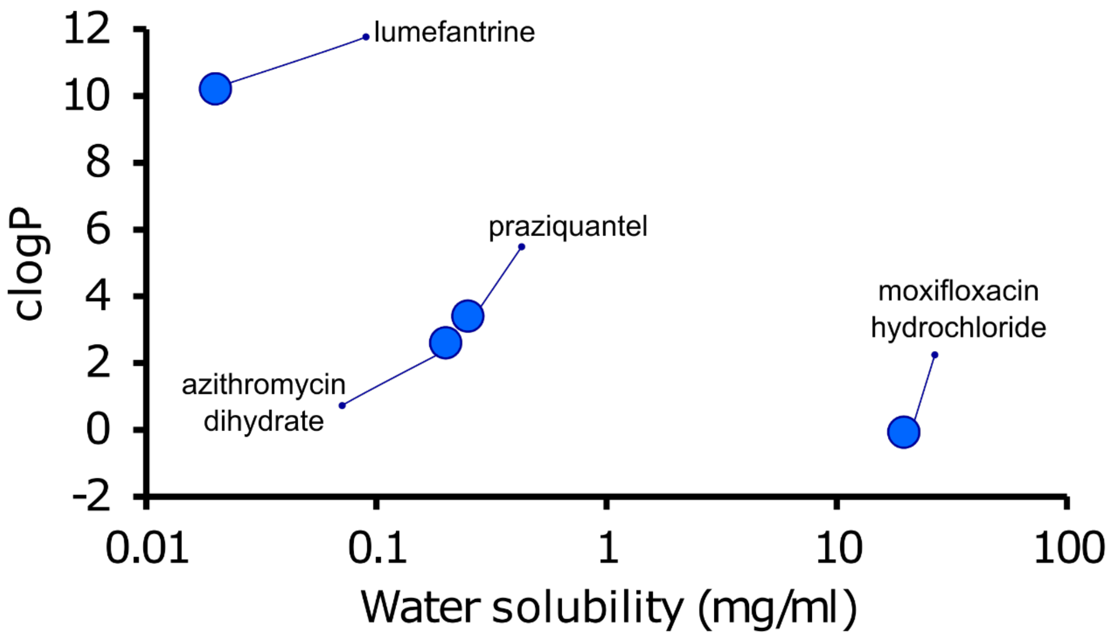

Supplementary figure 6: Solubility and calculated partition coefficients of the drugs used in this study

Supplementary Excel file contents

Data S1: Raw data used to make Figure 1. The caption used to describe Figure 1 is copied below:

Figure 1: Analysis of the World Health Organization's essential medicine list for children

(A) Relative proportion of drugs classified by disease area. **(B)** Detailed analysis of the relative proportion of drugs used for the treatment of pain, neurological and infectious diseases. **(C)** Routes of administration of drugs used for the treatment of infectious diseases. **(D)** Commonly used dosage forms for oral administration of anti-infective drugs.

Data S2: Raw data used to make Figure 2. The caption used to describe Figure 2 is copied below:

Figure 2: Physical characterization of oleogels

(A) Identification of gelling agents using an inversion assay. **(B)** Comparison of rheological properties of oleogels prepared using saturated fatty acids with differing carbon chain lengths. *$p<0.05$, One-way ANOVA, post-hoc Bonferroni versus palmitic acid at angular frequency = 10 rad/s. Data is represented as mean ± S.D., n = 3 **(C)** Comparison of rheological properties of oleogels prepared using saturated hydroxyl fatty acids. Data is represented as mean ± S.D., n = 3. *$p<0.05$, Student's t-test, versus 16-hydroxypalmitic acid at angular frequency = 10 rad/s. **(D)** Rheological analysis of oleogels prepared using stearic acid and stearyl alcohol. Data is represented as mean ± S.D., n = 3. *$p<0.05$, Student's t-test at angular frequency = 10 rad/s. **(E)** Rheological analysis of oleogels prepared using waxes. Data is represented as mean ± S.D., n = 3. **(F)** Differential scanning calorimetry analysis of oleogel containing ricebran wax as the gelling agent. Optical microscopy analysis of oleogels prepared using **(G)** hydroxystearic acid (scale bar = 400 μm) and **(H)** ricebran wax (scale bar = 200 μm).

Data S3: Raw data used to make Figure 3. The caption used to describe Figure 3 is copied below:

Figure 3: Analysis of drug solubility in oil-solubilizer library

(A) Composition of oils used in this study. **(B)** Chemical structures of solubilizers used in this study. Solubility of **(C)** azithromycin **(D)** praziquantel and **(E)** lumefantrine in oil-solubilizer libraries as measured using HPLC. Mean of three samples is reported in the heatmap.

Data S4: Raw data used to make Figure 4. The caption used to describe Figure 4 is copied below:

Figure 4: *In vitro* digestion and bioaccessibility studies

(A) Image of oleogel suspended in simulated salivary fluid **(B)** Images of oleogels in simulated gastric fluid and simulated intestinal fluid (fasted state). Insets on the right indicate cryo-TEM images of the intermediate phase in simulated intestinal fluid at those times. **(C)** HPLC quantification of release of praziquantel from oleogels in simulated salivary, gastric and intestinal fluids. Data is represented as mean ± S.D., n = 3 **(D)** Release of azithromycin from 36 oleogels was measured, and the area under the release curve was calculated. Heatmaps show mean area under the curve for three samples/formulation.

Data S5: Raw data used to make Figure 5. The caption used to describe Figure 5 is copied below:

Figure 5: Pharmacokinetics of oral and rectal oleogels in swine model

Concentration-time profiles of azithromycin in pigs dosed with **(A)** oral tablet **(B)** oral oleogel and **(C)** rectal oleogel are shown. The area under the curve (AUC) of the three formulations is shown in **(D)**. Concentration-time profiles of praziquantel in pigs dosed with **(E)** oral tablet **(F)** oral oleogel and **(G)** rectal oleogel are shown. The AUC of the three formulations is shown in **(H)**. Concentration-time profiles of lumefantrine in pigs dosed with **(I)** oral tablet **(J)** oral oleogel and **(K)** rectal oleogel are shown. The AUC of the three formulations is shown in **(L)**. For all pharmacokinetic curves, dotted lines indicate pharmacokinetics in individual animals and hard line

shows average pharmacokinetics. For all AUC assessments, open circles are individual data points, and bars indicate average. *indicates $p<0.05$, N.S. indicates that differences are not statistically significant, one-way ANOVA, post-hoc Bonferroni.

Data S6: Raw data used to make Figure 6. The caption used to describe Figure 6 is copied below:

Figure 6: *In vitro* and *in vivo* characterization of moxifloxacin oleopastes

Moxifloxacin oleopastes were synthesized and stored at **(A)** 4°C or **(B)** 40°C. Drug concentration in the top and bottom half of the pastes were measured at various time points. Open circles represent individual data points, and horizontal line indicates average value. N.S. indicates that differences are not statistically significant, one-way ANOVA, post-hoc Bonferroni. **(C)** Moxifloxacin oleopastes were stored at 4°C or 60°C for one week, following which drug concentrations in the top and bottom half of the paste were measured. Open circles represent individual data points, and horizontal line indicates average value. N.S. indicates that differences are not statistically significant, one-way ANOVA, post-hoc Bonferroni. Moxifloxacin was administered orally to swine as an **(D)** aqueous solution or **(E)** oleopaste, and systemic drug concentrations were measured. Dotted lines indicate pharmacokinetics in individual animals and hard line shows average pharmacokinetics (n = 3). **(F)** indicates AUC for animals treated with moxifloxacin aqueous solution and oleopaste. Open circles are individual data points, and bars indicate average. N.S. indicates that differences are not statistically significant, Student's t-test.

Data S7: Raw data used to make Figure 7. The caption used to describe Figure 7 is copied below:

Figure 7: Sensory evaluation of oleogels

(**A**) Flavor profiles of cottonseed oil, flaxseed oil and sesame oil. **(B)** Composition of oleogel formulations chosen for Stage 2 of testing. **(C)** Texture attributes and favorability of oleogels tested in Stage 2. **(D)** Texture map and **(E)** flavor map revealed from in-depth sensory analysis of nine oleogel formulations. Size the circles in (**D**) and (**E**) indicate the thickness of the gel.

Data S8: Raw data used to make Figure 8. The caption used to describe Figure 8 is copied below:

Figure 8: Macro-fluidic device for dosing of oleogels and oleopastes

(A) Images of single- and multi-dose devices for dispensing oleogels and oleopastes. **(B)** Quantification of dose dispensed from a single-dose device across three volunteers. **(C)** Measurement of dose dispensed from four pockets of the multidose dispenser **(D)** Comparison of dose dispensed from a multi-dose dispenser across three volunteers. Open circles are individual data points, and bars indicate average. N.S. indicates that differences are not statistically significant, one-way ANOVA.
